# Lactic Acid Bacteria Are Prevalent in the Infrabuccal Pockets and Crops of Ants That Prefer Aphid Honeydew

**DOI:** 10.3389/fmicb.2021.785016

**Published:** 2022-01-21

**Authors:** Zhou Zheng, Mengqin Zhao, Zhijun Zhang, Xin Hu, Yang Xu, Cong Wei, Hong He

**Affiliations:** ^1^Key Laboratory of National Forestry and Grassland Administration for Control of Forest Biological Disasters in Western China, College of Forestry, Northwest A&F University, Yangling, China; ^2^Key Laboratory of Plant Protection Resources and Pest Management, College of Plant Protection, Northwest A&F University, Yangling, China

**Keywords:** digestive tract, bacterial communities, ants, aphids, lactic acid bacteria

## Abstract

Ants are evolutionarily successful species and occupy diverse trophic and habitat niches on the earth. To fulfill dietary requirements, ants have established commensalism with both sap-feeding insects and bacteria. In this study, we used high-throughput sequencing of the bacterial 16S rRNA gene to characterize the bacterial composition and structure of the digestive tracts in three species of *Formica* ants and *Lasius niger* (Linnaeus)—species that predominantly feed on honeydew secreted by aphids. We found that bacterial communities displayed species- and colony-level signatures, and that bacterial communities in the infrabuccal pockets and crops were different from those in the midguts and hindguts. *Lactobacillus* and *Wolbachia* were dominant in the infrabuccal pockets and crops of workers, whereas *Wolbachia* was dominant in the midguts, hindguts and brood (larvae, pupae and cocoons). To learn more about the dominant *Lactobacillus* in ants, we assessed its prevalence in a wide range of aphid-tending ants using diagnostic PCR. We found that *Lactobacillus* was more prevalent in Formicinae than in Myrmicinae species. We also isolated four strains of lactic acid bacteria (*Lactobacillus sanfranciscensis*, *Lactobacillus lindneri*, *Weissella cibaria* and *Fructobacillus* sp.) from the infrabuccal pockets and crops of aphid-tending ants using a culture-dependent method. Two predominant lactic acid bacterial isolates, *Lactobacillus sanfranciscensis* (La2) and *Weissella cibaria* (La3), exhibited abilities in catabolizing sugars (sucrose, trehalose, melezitose and raffinose) known to be constituents of hemipteran honeydew. These findings contribute to further understanding the association between ants, aphids and bacteria, and provide additional information on the function of lactic acid bacteria in ants.

## Introduction

Ants are highly evolved social insects that live in organized colonies with labor division. The extraordinary abundance and species richness of ants have facilitated their evolutionary success in nature. To avoid food shortages, ants have evolved significant differentiation in feeding habits and related foraging behavior to utilize a variety of food resources. Ant species can be divided into predators, omnivores, functional herbivores, fungivores or scavengers, and can colonize a wide range of trophic and habitat niches (Hölldobler and Wilson, 1990; de Oliveira et al., 2016). One of the most interesting behaviors of ants is the habit of tending sap-feeding insects such as aphids and mealybugs throughout their 115–168 million years history (Currie et al., 1999; Stadler and Dixon, 2005; Moreau et al., 2006). Sap-feeding insects excrete carbohydrate-rich honeydew that serve as a food resource for natural enemies, e.g., ants and hyperparasitoids, and these insects defend the sap-feeders from predators and parasites as a reward (Way, 1963; Hölldobler and Wilson, 1990; van Neerbos et al., 2020).

Being plant-derived resources, honeydew contains complex sugars, amino acids, organic acid and some lipids, and the composition is depending on the host plant and sap-feeding insects themselves (Mittler, 1958; Hussain et al., 1974; Douglas, 1993; Fischer et al., 2002; Leroy et al., 2011; van Neerbos et al., 2020). It has been hypothesized that ants established a close association with bacteria to compensate for their imbalanced diets and diverse lifestyles. For instance, herbivorous Cephalotini ants harbor bacteria Rhizobiales, Burkholderiales, Pseudomonadales and Xanthomonadales that can metabolize essential and non-essential amino acids and recycle common nitrogenous wastes (Bution and Caetano, 2008; Russell et al., 2009; Hu et al., 2018). Omnivorous *Camponotus* ants contain intracellular symbiont *Blochmannia*, which supplies nitrogen and sulfur compounds for the host ants (Degnan et al., 2005; Gaudermann et al., 2006). Predatory ponerine ants (e.g., *Dinoponera*, *Neoponera*, *Pachycondyla* and *Odontomachus*) and army ants harbor *Mesoplasma*, *Spiroplasma*, *Wolbachia* and *Serratia*, which may be associated with their predatory lifestyle (Funaro et al., 2011; de Oliveira et al., 2016; Łukasik et al., 2017). These studies revealed that ants at different trophic levels harbor different types of bacteria, which are one of the major forces driving the dietary evolution and diversification of the host ants (Brady et al., 2006; Russell et al., 2009).

The digestive tract is an ideal place for bacterial colonization in ants as it contains abundant food. Moreover, different digestive regions exhibit different structures, functions and internal environments, which provide diet and habitats to various bacterial species. For instance, the ant *Tetraponera binghami* (Forel) has an elaborate pouch jutting from the anterior ileum, in which several types of bacteria are harbored (Billen and Buschinger, 2000; van Borm et al., 2002b). *Cataulacus* and *Cephalotes* ants harbor masses of bacteria in the posterior midgut and a dilated region of the anterior ileum (Caetano and da Cruz-Landim, 1985; Caetano et al., 1994). Certain *Formica* species house bacteria inside cubical cells that form a unicellular layer and are loosely arranged inside the perimeter of the midgut. Additionally, *Camponotus* ants carry endosymbionts *Blochmannia* in bacteriocytes intercalated with midgut epithelial cells (Schröder et al., 1996; Sauer et al., 2002).

Along with evolution, the discrepant feeding habits of ants have played a significant role in shaping the structure of their digestive tracts, especially the infrabuccal pocket (IBP) and proventriculus. The IBP is located in the preoral cavity of ant adults (Gotwald, 1969; Richter et al., 2019), which mainly serves as a filter to obstructs solid particles and allows only fluids and sufficiently small particles to flow into the digestive tube (Eisner and Happ, 1962; Gotwald, 1969). The specialized hairs inside the pocket are helpful in filtering solid pellets and bacteria (Wang et al., 2018, 2019). A recent study on the bacterial communities of two predatory ants, *Odontomachus monticola* Forel and *Ectomomyrmex javanus* Mayr, has shown that although bacterial communities differ significantly between the IBPs and guts of both ant species, they are similar in IBPs of workers and larvae in *O. monticola*. This provides meaningful information regarding the function of the IBP in ants (Zheng et al., 2021). The IBP can also prevent the invasion and spread of the microbial parasite *Escovopsis* in the workers of leaf-cutting ants (Little et al., 2003, 2006).

Studies on the proventriculus of ants were more systematical when compared to those on the IBP. In general, formicines and dolichoderines exhibit a plesiomorphic and highly elaborated proventriculus with occlusory tract or sclerotized cupola, which can obstruct the passage of large solid particles to the midgut (Eisner, 1957). However, in most myrmicines, the proventriculus is comprised of a simple tube and sphincter, which is different from that of formicines and dolichoderines (Eisner, 1957). Studies on *Cephalotes* have revealed the effect of the proventriculus in shaping bacterial communities, of which the evolved proventriculus possessing sclerotized structures (Urbani and De Andrade, 1997) that can obstruct the passage of microspheres larger than 0.2 μm, thus leading to a variation in the bacterial communities of the crop and midgut (Lanan et al., 2016). These studies provide a crucial viewpoint, suggesting that the structure of the digestive tract is a key factor that shapes the bacterial communities of ants. However, studies of the bacterial communities in the crop and midgut of ants are relatively rare.

To date, few studies have combined the investigation of bacterial composition and diversity with the ecology, behavior, and environmental conditions of ants, so this limits our understanding of the factors that affect the associations between ants and microbe. In addition, most investigations used the whole guts or bodies of ants as objectives, which leave blind spots in understanding the relationship between bacterial variation and the morphological structure of different digestive regions in ants. Moreover, there is limited knowledge regarding the functions of bacterial communities in the digestive tracts of ants, and this merits further exploration.

*Formica* and *Lasius*—two common genera belonging to the subfamily Formicinae, are generally omnivorous. Their diet is mainly derived from two sources: the honeydew produced by aphids and other sap-sucking insects (which is used as an energy source for metabolic maintenance), and the insect prey and scavenged materials that is used to provide proteins for brood rearing (Lange, 1960). In addition, seeds, tree sap and berry juices are also used as food sources by these ants. *Formica japonica* Motschoulsky, *F. sanguinea* Latreille, *F. gagatoides* Ruzsky, and *Lasius niger* (Linnaeus) are four geographically widespread ant species in the Qinling Mountains, China, and form mutualistic “food-protection” relationships with some sap-sucking insects. This makes them an ideal study system to analyze the gut bacterial composition and diversity of honeydew-feeding ants.

In the present study, we investigated the composition and structure of bacterial communities in the digestive tracts of these four ant species through sequencing the V3–V4 regions of the bacterial 16S rRNA gene. We further investigated the distribution of *Lactobacillus* in more honeydew-feeding ant species through diagnostic PCR. In addition, we isolated several strains of lactic acid bacteria and assessed their ability in degrading sugars. Our study provides macro and micro perspectives in understanding the bacterial communities in honeydew-feeding ants, particularly the association between bacterial community and morphologically differentiated digestive regions in ants and the commensal relationship between bacteria and related sap-feeding insects.

## Materials and Methods

### Ant Collection, Dissection, and DNA Extraction for High-Throughput Sequencing

A total of ten colonies of formicines representing four species (*F*. *japonica*, *F. sanguinea*, *F*. *gagatoides* and *L*. *niger*) were collected from Ningshan County, Taibai County, and Xi’an City in Shaanxi Province, China from June to August in 2018 and 2019 (Table 1). *F. japonica* and *F. sanguinea* were collected from three different colonies, respectively, and *F. gagatoides* and *L. niger* were collected from two different colonies, respectively. Workers were included in all colonies; however, brood (larvae, pupae and cocoons) were obtained occasionally during collection. These samples were used to investigate the effects of species, colonies and developmental stages of ants on the bacterial communities.

**TABLE 1:** Information of 168 samples from the four formicine ant species in this study.

Ant species	Colony IDs	Samples	Collection sites and nesting habits	Longitude and latitude
*Formica japonica* Motschoulsky	Fj1	Fj1WI (IBPs), Fj1WC (crops); Fj1WM (midguts), Fj1WH (hindguts), Fj1L (larvae), Fj1P (pupae), Fj1C (cocoons)	Ningshan County, nesting in soil	33°34′11.4″ N 108°31′12.9″ E
	Fj2	Fj2WI (IBPs), Fj2WC (crops), Fj2WM (midguts), Fj2WH (hindguts), Fj2L (larvae), Fj2P (pupae), Fj2C (cocoons)	Ningshan County, nesting in soil	33°39′88.6″ N 108°36′91.1″ E
	Fj3	Fj3WI (IBPs), Fj3WC (crops), Fj3WM (midguts), Fj3WH (hindguts), Fj3P (pupae), Fj3C (cocoons)	Taibai County, nesting in soil	34°21′35.3″ N 107°14′13.6″ E
*Formica sanguinea* Latreille	Fs1	Fs1WI (IBPs), Fs1WC (crops); Fs1WM (midguts), Fs1WH (hindguts), Fs1L (larvae), Fs1P (pupae), Fs1C (cocoons)	Ningshan County, nesting in soil	33°34′11.4″ N 108°31′12.9″ E
	Fs2	Fs2WI (IBPs), Fs2WC (crops), Fs2WM (midguts), Fs2WH (hindguts)	Ningshan County, nesting in soil	33°39′88.6″ N 108°36′91.1″ E
	Fs3	Fs3WI (IBPs), Fs3WC (crops), Fs3WM (midguts), Fs3WH (hindguts), Fs3L (larvae), Fs3P (pupae), Fs3C (cocoons)	Taibai County, nesting in soil	34°21′35.3″ N 107°14′13.6″ E
*Formica gagatoides* Ruzsky	Fg1	Fg1WI (IBPs), Fg1WC (crops), Fg1WM (midguts), Fg1WH (hindguts), Fg1P (pupae)	Ningshan County, nesting in soil	33°47′12.4″ N 108°49′64.4″ E
	Fg2	Fg2WI (IBPs), Fg2WC (crops), Fg2WM (midguts), Fg2WH (hindguts), Fg2L (larvae), Fg2P (pupae), Fg2C (cocoons)	Xi’an City, nesting in wood	33°78′11.9″ N 108°57′70.1″ E
*Lasius niger* (Linnaeus)	Ln1	Ln1H (heads), Ln1A (abdomans), Ln1L (larvae)	Taibai County, nesting in soil	34°06′84.5″ N 107°30′04.6″ E
	Ln2	Ln2H (heads), Ln2A (abdomans), Ln2L (larvae)		34°03′74.6″ N 107°24′14.2″ E

The workers and broods were stored in buckets, brought back to laboratory, and only fed with sterile water for approximately 48 h before vivisection. An individual *Formica* ant worker was narcotized at –20°C for 3 min, surface sterilized by rinsing with 70% ethanol for 2 min, then washed with sterile ddH_2_O several times. It was then placed on a sterile glass slide, the gaster was separated from the head, and the cuticular membrane was removed with sterile ultra-fine forceps. Following this, the whole gut was exposed, the crop, midgut and hindgut were separated with sterilized forceps, and each part was washed with sterile ddH_2_O at least three times and placed in separated 1.5 mL centrifuge tubes. For dissection of the IBP, the cuticular membrane of the dorsal head was removed, and the IBP was exposed and clamped with sterile ultra-fine forceps, and placed in a 1.5 mL centrifuge tube. Sterilized forceps were flame sterilized between every change in the IBP and gut regions during the dissections to avoid cross-contamination, and all procedures were conducted on a clean bench. We pooled each part of digestive tract from ten workers from each colony to acquire sufficient DNA for sequencing. Due to the small body size of *L. niger* workers (size < 5 mm), we could not dissect out the digestive tract; therefore, the heads and gasters from 5 workers were pooled, respectively. Larvae, pupae and cocoons were surface sterilized by rinsing with 70% ethanol for 2 min, washed with sterile ddH_2_O several times, and placed in sterile 1.5 mL centrifuge tubes. We pooled the samples of different development stages from three individuals. All samples were stored in –80°C until DNA extraction.

A total of 168 samples were obtained from different colonies of the four ant species: *F. japonica* (Fj1, 21 samples; Fj2, 21 samples; and Fj3, 18 samples), *F. sanguinea* (Fs1, 21 samples; Fs2, 12 samples; and Fs3, 21 samples), *F. gagatoides* (Fg1, 15 samples; and Fg2, 21 samples), and *L. niger* (Ln1, nine samples; and Ln2, nine samples). As negative controls for removing possible bacterial contamination during dissection, DNA was extracted from sterile ddH_2_O, and ddH_2_O rinsed off from surface sterilized ants, each digestive section, and the IBPs. A total of 12 negative control samples were included.

All samples were homogenized using sterile pestles and incubated in lysozyme for 30 min to lyse the cell walls of Gram-positive bacteria (Segers et al., 2019). Genomic DNA was extracted using the DNeasy Blood & Tissue kit (Tian Gen, Beijing, China) following the manufacturer’s instructions. The extracted DNA was dissolved in 50 μL TE buffer. The concentration and purity of the extracted DNA was quantified using NanoDrop 1000 Microvolume Spectrophotometers (Thermo Fisher Scientific, Waltham, MA, United States). Qualified samples were stored at –80°C until sequencing.

### Bacterial 16S rRNA Gene Amplification and High-Throughput Sequencing

DNA extractions of 168 ant samples and 12 negative controls were sent to the Biomarker Technologies Corporations (Beijing, China) for high-throughput sequencing. All samples were sequenced using the Illumina HiSeq 2500 platform. The primer pair 338F (5′-ACTCCTACGGGAGGCAGCA-3′) and 806R (5′-GGACTACHVGGGTWTCTAAT-3′) was used to amplify the V3–V4 hypervariable region of the bacterial 16S rRNA gene (Anahtar et al., 2016). The PCR was conducted in a total reaction volume of 50 μL containing 10 μL 5 × Q5 Reaction buffer, 0.5 μL Q5 High-Fidelity DNA polymerase (2 U/μL) (TaKaRa, Dalian, China), 10 μL High GC Enhancer, 1 μL dNTP mixture (10 mM), 5 μL per primer (10 μM), 60 ng genomic DNA, and nuclease free water was added to make up a total volume of 50 μL. After an initial denaturation at 95°C for 5 min, the amplification was performed for 15 cycles of denaturation at 95°C for 1 min, annealing at 50°C for 1 min, and extension at 72°C for 1 min, followed by a final extension at 72°C for 7 min. The amplified products were detected using the 1.8% agarose gel electrophoresis method and purified using Vahtstm DNA Clean Beads (Vazyme, Nanjing, Jiangsu, China). A second round of PCR then performed in a 50 μL reaction volume that containing 10 μL 2 × Phusion HF MM, 5 μL per primer (10 μM), 10 μL PCR products from the previous step, and nuclease free water was added to make up a total volume of 50 μL. The thermal cycling conditions for the second PCR consisted of initial denaturation at 98°C for 30 s, followed by 10 cycles of denaturation at 98°C for 10 s, annealing at 65°C for 30 s, and extension at 72°C for 30 s, followed by a final extension at 72°C for 5 min. Finally, all PCR products were quantified using the Quant-iT™ dsDNA HS Reagent and pooled together. Although no PCR products were detected in the negative controls, these samples were run on the Illumina HiSeq 2500 platform to detect contaminants that may have been present in low abundance or other potential contaminants derived from the environment and during process of DNA extraction and sequencing.

The V3–V4 hypervariable region of the bacterial 16S rRNA gene was too short for further investigation. Therefore, we sequenced the full-length 16S rRNA gene of 14 samples representing the IBPs and crops of *Formica* ants and 2 samples representing the heads and abdomens of *L. niger* using the PacBio SMRT platform. The universal primer pair 27F (5′-AGRGTTTGATYMTGGCTCAG-3′) and 1492R (5′-GGYTACCTTGTTACGACTT-3′), each one labeled with a 16-nucleotide barcode, was used for full length 16S rRNA amplification (Frank et al., 2008). The PCR reaction volume was conducted in 50 μL volume containing 25 μL 2 × KOD FX Neo buffer, 1 μL KOD FX Neo polymerase (1.0 U/μL) (TaKaRa, Dalian, China), 10 μL dNTP mixture (2 mM), 1.5 μL per primer (10 μM), and 60 ng genomic DNA. The thermocycling program was: 95°C for 5 min, followed by 30 cycles at 95°C for 30 s, 50°C for 30 s, and 72°C for 1 min, and a final extension at 72°C for 7 min. The PCR products were purified according to the protocol of Biomarker Technologies Corporations (Beijing, China). The PCR products were quantified and checked using an Agilent DNA 1000 Kit and an Agilent 2100 Bioanalyser (Agilent Technologies, Santa Clara, CA, United States) according to the manufacturer’s instructions. The purified amplification products were sequenced using the P6-C4 chemistry on a PacBio RS II instrument (Pacific Biosciences, Inc., Menlo Park, CA, United States).

### Bioinformatics Analysis

Recently, new methods have been developed for bioinformatic analysis of sequences derived from high-throughput sequencing. In particular, amplicon sequence variants (ASVs) are explicitly designed to replace operational taxonomic units (OTUs), so as to cluster sequences without imposing an arbitrary similarity threshold (Callahan et al., 2017). We used the ASVs methods to process the sequences of the bacterial 16S rRNA gene.

After sequencing, raw tags were quality filtered by Trimmomatic (v. 0.33) to obtain clean tags (Bolger et al., 2014). The primer sequences were identified and removed with Cutadapt 1.9.1 to obtain high quality reads, and the overlapping regions between high quality reads were merged into clean reads using Flash v1.2.7 (Magoč and Salzberg, 2011). The reads were then denoised and chimeras were removed with the Data2 in Qiime2 (Callahan et al., 2016; Bolyen et al., 2019), following which we conducted feature classification to output an ASVs table. The ASVs were clustered and assigned into taxonomic groups by searching against the SILVA database with a naive Bayes classifier (Quast et al., 2013). Negative controls were filtered using the Decontam package based on the frequency method in R v4.0.3 following default settings (Davis et al., 2018). After the feature table was filtered, ASVs classified as chloroplasts, mitochondria and archaeal ASVs were deleted in the Qiime2 and only bacterial ASVs were retained.

To clearly visualize the differences of bacterial communities, samples were divided into three groups: species, colonies, and digestive regions + development stages. To identify bacterial genera with significant difference in relative abundance within groups, we conducted biomarker discovery with linear discriminant analysis effect size (LEfSe) analysis based on a non-parametric Kruskal–Wallis rank sum test in MicrobiomeAnalyst,^1^ and conducted discriminant analysis to evaluate the effect size of those significant genera (Dhariwal et al., 2017). The *p*-value was adjusted to account for the false discovery rate, and a log LDA score of 2 was used as the cutoff value. Bacterial genera with adjusted *p*-value < 0.05 were regarded as having significantly different abundance between the compared groups. Beta diversity analyses of different groups were performed with the q2-diversity plugin in Qiime2. Non-metric multidimensional scaling (NMDS) was performed in Qiime2 based on the Bray–Curtis distance to assess bacterial structures between groups. Statistical differences between the beta diversity metrics of groups were analyzed with the analysis of similarities (ANOSIM) test using the vegan package in Qiime2 based on the Bray–Curtis distance with 999 permutations.

The procedures used to analyze bacterial 16S rRNA full-length gene were different from those used to analyze partial 16S rRNA gene. After sequencing, raw subreads were calibrated with SMRT Link v8.0 to acquire circular consensus sequences (CCS). CCS were identified by LIMA v1.7.0 based on barcode sequences, the sequences length was filtered by PRINSEQ v.0.20.4 (Schmieder and Edwards, 2011), and the chimeric sequences were identified and removed by UCHIME v8.1 to obtain high quality CCS. The barcodes and primer sequences of high-quality sequences were removed, and these sequences were denoised with Data2 in Qiime2 (Callahan et al., 2016; Bolyen et al., 2019). Finally, the ASVs were clustered and assigned into taxonomic groups by searching against the SILVA database with a naive Bayes classifier (Quast et al., 2013).

### Detection of *Lactobacillus* and Uncultured Acetobacteraceae Using Diagnostic PCR

Ant workers that tending aphids were collected from 53 sites distributed across Xi’an City, Qian County, Taibai County and Ningshan County. These ant workers represented 15 species, six genera, and three subfamilies (Supplementary Table 1). From each sampling site, 10 workers were collected and brought back to the laboratory after preserving in absolute ethyl alcohol. All workers were surface sterilized by rinsing in 70% ethyl alcohol and then washed with sterile ddH_2_O. DNA was extracted from individual ants using the DNeasy Blood & Tissue Kit (Tian Gen, Beijing, China) according to the manufacturer’s instructions.

The specific primers L-F1 (5′-AGCAGTAGGGAATCTTCCA-3′) and L-R1 (5′-CACCGCTACACATGGAG-3′) were used to determine the distribution of *Lactobacillus* in each worker (Heilig et al., 2002; Nielsen et al., 2003). The PCR reaction was conducted in 25 μL volume containing 12.5 μL 2 × Rapid Taq Master Mix (Vazyme, Nanjing, China), 1 μL of each primer (10 μM), and 1 μL genomic DNA. The thermocycling program was as follows: initial denaturation at 95°C for 5 min, followed by 35 cycles of denaturation at 95°C for 30 s, annealing at 55°C for 30 s, and extension at 72°C for 30 s, and followed by a final extension at 72°C for 7 min.

### Isolation and Identification of Lactic Acid Bacteria Strains From Honeydew-Feeding Ants

For isolation of *Lactobacillus* and other lactic acid bacteria from foraging workers, ants were collected from the campus of Northwest N&F University in Yangling, Shaanxi Provence, China, in August of 2019. Workers that frequently moved back and forth between their nests and trees infected with plentiful aphids were chosen. Of the seven ant species, four were dissected into heads and gasters due to their minute body size, pooling 3 individuals of each colony as one sample. The remaining three species were dissected under sterile conditions (as described above) to obtain IBPs, crops, midguts and hindguts, pooling 5 individuals of each colony to compose a sample (Supplementary Table 2). A diluted sample (100 μL) was plated on Man-Rogosa Sharpe agar plates (MRS, Beijing, China) and supplemented with sterile actinone (40 mg/L) to restrain the growth of other bacteria. The inoculated plates were cultured in an anaerobic condition at 30°C for 48–72 h. The anaerobic condition was created using anaerobic jars and CO_2_ gas packs (Sanling, Darmstadt, Japan). Colonies of lactic acid bacteria with different morphology (color, shape and size) were counted and picked up, and single representative colonies were streaked and propagated on MRS agar mediums as described above. The pure isolates were propagated in MRS broth and preserved in glycerin at –80°C.

For bacterial identification, genomic DNA was extracted from the selected isolates using the DNeasy Blood & Tissue kit (Tian Gen, Beijing, China) following the manufacturer’s instructions. PCR amplification of the whole 16S ribosomal RNA gene was performed with the universal primers 27F and 1492R (Frank et al., 2008). The amplification was performed in 25 μL containing 2 μL dNTP mixture, 2.5 μL 10 × Taq buffer, 0.25 μL (5 U/μL) Taq DNA polymerase (TaKaRa, Dalian, China), 1 μL of each primer (10 μM), and 1 μL of genomic DNA. PCR amplification parameters were: 95°C for 5 min, followed by 30 cycles at 95°C for 30 s, 55°C for 30 s and 72°C for 2 min, and a final extension at 72°C for 10 min. The PCR products were detected on a 1% agarose gel, then they were purified with TIAN Midi Purification kit (Tian Gen, Beijing, China) following manufacturer instructions and pair-end Sanger sequenced in Sangon Biotech (Shanghai, China). The sequences obtained were assembled with the SeqMan program and BLAST-searched in the National Center for Biotechnology Information (NCBI) GenBank.

Phylogenetic analysis was performed to establish the evolutionary relationships among the isolate strains of *Lactobacillus* in this study and other lactic acid bacteria. A maximum-likelihood phylogenetic tree was constructed using the MEGA v7.0 software (Kumar et al., 2016). The best fit model was GTR, and the strength of tree topologies was verified through 2000 bootstrap replicates.

### Measurements of Sugar Catabolism Ability of Isolated Lactic Acid Bacterial Strains

Two predominant isolated lactic acid bacteria, *Lactobacillus sanfranciscensis* (La2) and *Weissella cibaria* (La3), were used in assessing the sugar catabolic ability. Pure isolates of the two strains were inoculated in 10 mL MRS broth (Land Bridge, Beijing, China) and incubated at 30°C for 48 h. Following this, 2 mL of each bacterial inoculation was added into 50 mL MRS broth and cultured at 30°C for 48 h, respectively, and 30 mL culture liquid was centrifuged at 8,000 × g for 5 min at room temperature to harvest bacteria, and these bacteria were suspended and washed twice in sterile water.

The sugar catabolic ability of each strain was determined by mixing 1 mL bacterial suspension with 9 mL MRS broth (without glucose), and 1 mL 0.2% (w/v) sugars (sucrose, melezitose, trehalose, and raffinose) were added into the mixture, respectively. Samples in which the bacteria were inactivated by boiling water, and samples in which addition of sugars was omitted, served as two types of negative controls. Then, all samples were incubated at 30°C for 6, 12, and 24 h consecutively.

Subsequently, 200 μL mixture of each time point was added with 1 mL of 80% (v/v) ethanol, and were incubated at 70°C for 1 h. After the mixture cooled to room temperature, it was centrifuged at 12,000 × g for 20 min. The supernatant was transferred to a 1.5 mL centrifuge tube, and incubated at 95°C until the ethanol was evaporated. The remaining solution was desiccated at 50°C to obtain dry sugar extracts. Sugar extracts were dissolved with 100 μL sterile double-distilled water, and filtered through a 0.22 μm membrane, then subjected to High-Performance Liquid Chromatography (HPLC) analysis with minor revision to detect the contents of sugars (Wang et al., 2003). Specifically, the Waters Xbridge™ amide column (3.5 μm, 4.6 × 250 mm) was washed using acetonitrile/0.02% Ammonia solution (67:33, v/v) as a mobile phase at 0.6 mL/min for the separation of soluble carbohydrate components. The constitutive sugar values of negative controls were subtracted from samples. An evaporative light-scattering detector (ELSD, Waters 2424) was applied to monitor the sugar signal.

## Results

### Characterization and Taxonomic Classification of Bacterial Communities in Partial 16S rRNA Gene Sequences

In total, 12,067,636 raw tags were generated based on sequencing the V3–V4 regions of the 16S rRNA gene for all samples, of which 10,701,931 effective tags were retained after quality filtering. The average length of each sample ranged from 407 to 427 bp. A total of 944 ASVs were generated across all samples. Rarefaction curves of each sample increased slowly, suggested that majority of bacteria in each sample were included (Supplementary Figure 1).

**FIGURE 1:**
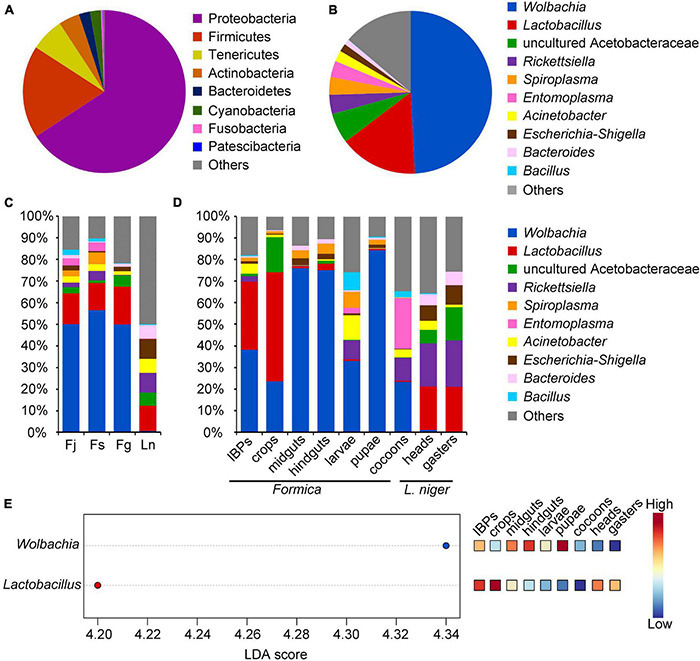
Relative abundance of bacteria (phyla and genera) found in four ant species sequenced by Illumina HiSeq 2500 platform, and significant different bacteria across samples. **(A)** Abundance of bacteria showing phyla with more than 1% relative abundance in all samples. **(B)** Abundance of bacteria showing genera with more than 1% relative abundance in all samples. **(C)** Abundance of bacterial genera across each ant species. **(D)** Abundance of bacterial genera across different digestive tract regions and broods of three *Formica* ant species, and in heads and gasters of *L. niger*. **(E)** Bacterial genera that significantly differ (FDR adjusted *p*-value < 0.05) in abundance across different digestive tract regions and broods of three *Formica* ant species, and heads and gasters of *L. niger*. Significant bacterial genera were ranked in decreasing order based on their LDA score (*x*-axis). The mini heatmap to the right of the plot indicated whether the relative abundance of bacterial genera was higher (red) or lower (blue) in each group.

The ASVs were annotated into eight bacterial phyla, of which Proteobacteria (65.74%) and Firmicutes (18.41%) were the dominant phyla, followed by Tenericutes (6.63%), Actinobacteria (4.17%), Bacteroidetes (2.22%), Cyanobacteria (2.16%), Fusobacteria (0.36%), and Patescibacteria (0.11%) (Figure 1A). At the genus level, nine bacterial genera were identified, of which *Wolbachia* (48.19%) and *Lactobacillus* (15.19%) were the dominant genera, followed by uncultured Acetobacteraceae (5.92%), *Rickettsiella* (3.74%), *Spiroplasma* (3.46%), *Entomoplasma* (3.17%), *Acinetobacter* (2.14%), *Escherichia*-*Shigella* (1.57%), and *Bacteroides* (1.06%) (Figure 1B).

Taxonomic analysis showed that the bacterial genera varied across different ant species, digestive tract regions, and broods (Figures 1C,D). Specifically, the three *Formica* ant species exhibited similar bacterial communities that were dominated by *Wolbachia* and *Lactobacillus* (Figure 1C). *L. niger* possessed different dominant bacteria compared to *Formica* ants, and included *Lactobacillus*, uncultured Acetobacteraceae, *Rickettsiella*, *Acinetobacter*, and *Escherichia*-*Shigella* (Figure 1C). Moreover, LefSe analysis showed that the abundance of *Wolbachia* was significantly higher in *Formica* ants than in *L. niger* (*P* < 0.05), whereas the abundances of other bacteria were not statistically different among ant species (*P* > 0.05).

Among the different digestive tract regions and broods of *Formica* ants, the IBPs and crops had similar bacterial communities dominated by *Wolbachia* and *Lactobacillus*; however, uncultured Acetobacteraceae was more abundant in the crops than in the IBPs (Figure 1D). Unlike the IBPs and crops, the midguts and hindguts were both dominated by *Wolbachia* (Figure 1D). Bacterial genera between the brood (larvae, pupae and cocoons) of *Formica* ants showed that the larvae and cocoons had similar bacterial communities dominated by *Wolbachia* and *Rickettsiella*. However, they also presented some differences in bacterial communities, such as that *Acinetobacter* and *Spiroplasma* were more abundant in the larvae, and *Entomoplasma* was more prevalent in the cocoons. *Wolbachia* was dominant in the pupae, which is similar to the midguts and hindguts. LefSe analysis showed that the abundance of *Wolbachia* was significantly higher in the midguts, hindguts and pupae than in other samples (Figure 1E), and that the abundance of *Lactobacillus* was significantly high in the IBPs and crops of *Formica* ants (Figure 1E).

### Structures of Bacterial Community Across Different Species, Colonies, Digestive Tract Regions, and Broods of Four Ant Species

**FIGURE 2:**
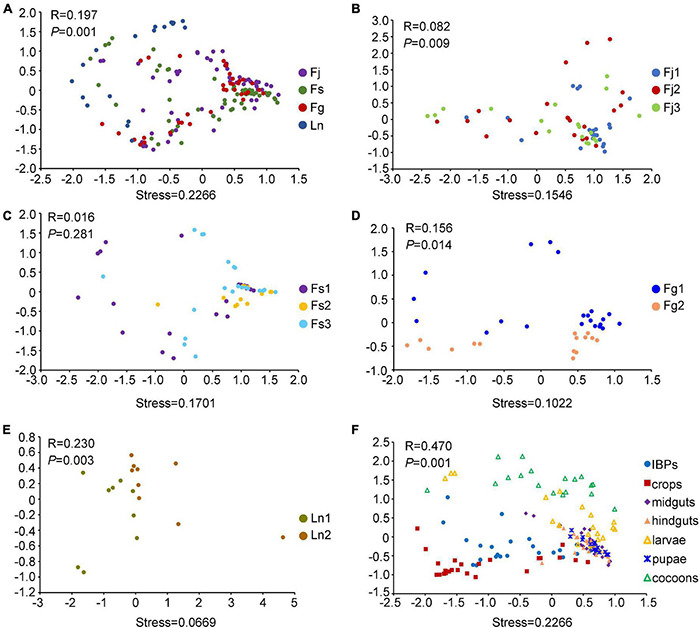
Non-metric Multidimensional scaling (NMDS) analysis (Bray-Curtis distance) showing differences in bacterial structures. **(A)** NMDS analysis of bacterial structures across four ant species. **(B)** NMDS analysis of bacterial structures across three colonies (Fj1, Fj2, Fj3) of *F. japonica*. **(C)** NMDS analysis of bacterial structures across three colonies (Fs1, Fs2, Fs3) of *F. sanguinea*. **(D)** NMDS analysis of bacterial structures across two colonies (Fg1, Fg2) of *F. gagatoides*. **(E)** NMDS analysis of bacterial structures across two colonies (Ln1, Ln2) of *L. niger*. **(F)** NMDS analysis of bacterial structures across different digestive tract regions and brood (larvae, pupae, and cocoons) of three *Formica* ant species. Dots in different color represented different groups. PERMANOVA tests showed statistical analysis between groups.

NMDS plots showed that the bacterial community structures were similar among the three *Formica* ant species, but differed slightly with *L. niger* (Figure 2A). ANOSIM analyses showed that the four ant species differed significantly from each other in beta diversity metrics (*R* > 0, *P* < 0.001). Apart from differences between ant species, the bacterial community structures also differed between colonies within each species, except in the colonies of *F*. *sanguinea* (Figures 2B–E). Moreover, their beta diversity metrics differed significantly across colonies as well (*R* > 0, *P* < 0.05). The contribution of digestive tract regions and broods to the community structures was obvious: the IBPs and crops of the three *Formica* ant species clustered together (Figure 2F), and the midguts, hindguts, pupae and most larvae samples clustered tightly with each other (and separately from cocoons), and their beta diversity metrics differed significantly (*R* > 0, *P* < 0.05) (Figure 2F).

### Full-Length 16S rRNA Gene of Representative Samples From IBPs and Crops

The results of high-throughput sequencing of the V3–V4 regions of the bacterial 16S rRNA gene revealed that, the bacterial communities in the IBPs and crops of the three *Formica* species were similar and dominated by *Lactobacillus* and *Wolbachia*. However, the sequences of these bacteria were too short for further investigation; therefore, 16 samples from the IBPs and crops of *Formica* ants, heads and gasters of *L. niger* were processed in the PacBio SMRT platform to acquire the full-length 16S rRNA gene sequences of the bacterial communities.

**FIGURE 3:**
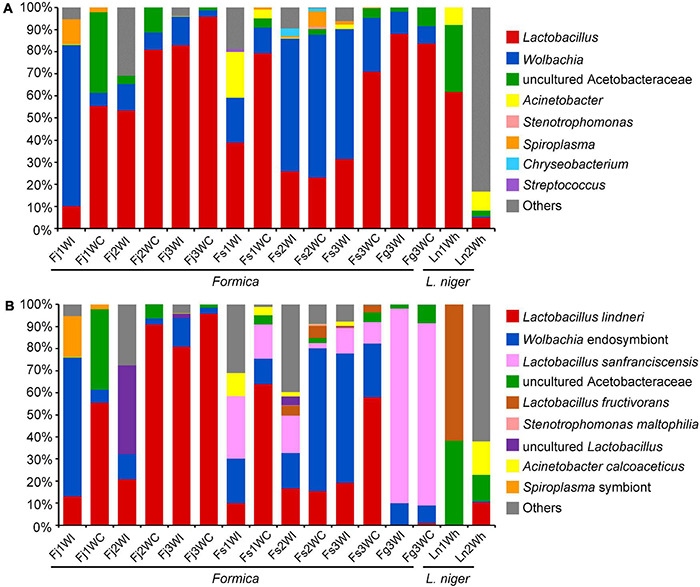
Relative abundance of bacteria found in the IBPs and crops of *Formica* ants, heads and gasters of *L. niger* sequenced by PacBio SMRT platform. **(A)** Abundance of dominant bacterial genera. **(B)** Abundance of bacterial species.

A total of 119,040 CCS were obtained for all samples with an average read length of 1,500 bp. Taxonomic analysis indicated that bacterial ASVs were classified into eight dominant bacterial genera: *Lactobacillus*, *Wolbachia*, uncultured Acetobacteraceae, *Acinetobacter*, *Stenotrophomonas*, *Spiroplasma*, *Chryseobacterium*, and *Streptococcus* (Figure 3A). The bacterial genera in these samples were similar to the results acquired by sequencing the V3–V4 region of 16S rRNA gene (Figure 1B).

**FIGURE 4:**
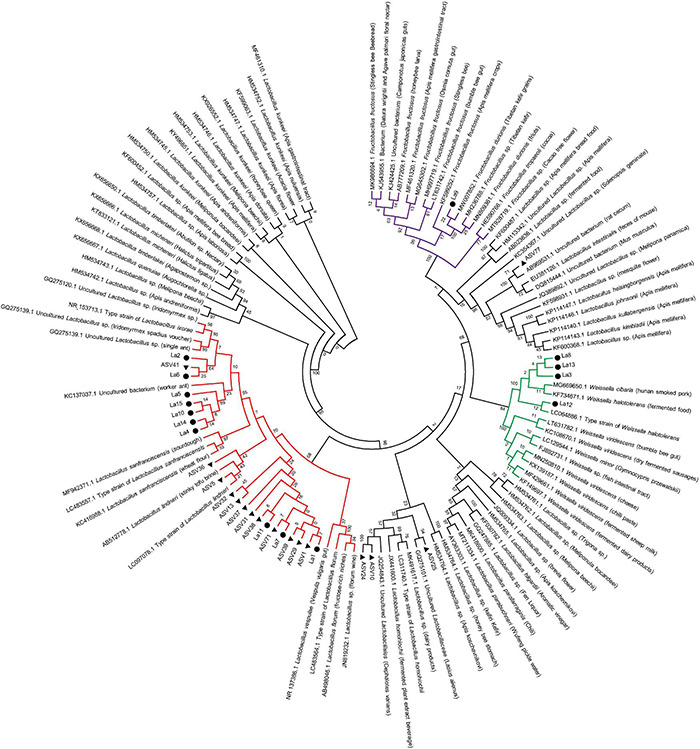
Phylogenetic circle tree based on a distance matrix of 16S rRNA genes from 15 isolated strains of lactic acid bacteria, representative ASVs of *Lactobacillus*, and their most closely related bacterial sequences in NCBI. The phylogenetic tree was constructed by ClustalW using the maximum-likelihood method within the MEGA 7.0 package. Closely related sequences were shown together with accession numbers (in parentheses) from GenBank. Bootstrap value based on 2,000 replication displayed the significance of the interior nodes, and are shown at branch points. Solid black dots, isolated lactic acid bacteria strains; solid black triangle, respective *Lactobacillus* ASVs. Branches in different color represents groups of lactic acid bacteria.

Using the full-length 16S rRNA gene sequences, several bacteria were annotated to the species level, including *Lactobacillus lindneri*, *Lactobacillus sanfranciscensis*, *Lactobacillus fructivorans*, *Acinetobacter calcoaceticus*, and *Stenotrophomonas maltophilia* (Figure 3B). However, many ASVs could not be annotated into explicit species. A total of 47 representative ASVs of *Lactobacillus* were identified. Of these, 28 ASVs were annotated into *Lactobacillus lindneri*, which was dominant in the crops of *F. japonica* and *F. sanguinea*. Moreover, 14 ASVs were annotated as *Lactobacillus sanfranciscensis*, which was dominant in the IBPs and crops of *F. gagatoides*. Three ASVs were assigned to *Lactobacillus fructivorans*, which was dominant in the heads of *L. niger*. Two ASVs were assigned to uncultured *Lactobacillus*, which was dominant in the IBPs from the Fj2 colony of *F. japonica*. Ten representative ASVs of uncultured Acetobacteraceae were identified, but could not be annotated into any species. The phylogenetic tree showed that *Lactobacillus* ASVs were closely related to several *Lactobacillus* species that are common inhabitants in fermented materials, bees, and ants but are not specific to them (Figure 4).

### Prevalence and Distribution of *Lactobacillus* in Other Honeydew-Feeding Ants

To further investigate the prevalence and distribution of *Lactobacillus*, we acquired ∼1,500 bp of full-length 16S rRNA gene sequences of respective samples. Then, we applied diagnostic PCR to a wide variety of ants that feed on aphid honeydew.

The results showed that *Lactobacillus* was prevalent in 12 of the 15 examined ant species from 53 sampling sites (Supplementary Table 1). The frequency of *Lactobacillus* infections was high in the six *Formica* ant species, including *F. japonica* (84.44%, nine sites), *F. glauca* Ruzsky (62.22%, nine sites), *F. cunicularia* Latreille (72.73%, 11 sites), *F. gagatoides* (80%, one site), *F. sanguinea* (90%, one site), and *F. polyctena* Foerster (100%, one site), respectively (Table 2). The infection rates of *Lactobacillus* varied greatly in *Lasius* and *Camponotus* ants, including *L. niger* (32.86%, 14 sites) *L. fuliginosus* (Latreille) (100%, one site), *C. japonicus* (42.86%, seven sites), and *C. obscuripes* Mayr (60%, one site) (Table 2). The infection rates of *Lactobacillus* in *Pristomyrmex pungens* Mayr was 100% (one site) (Table 2). However, the *Lactobacillus* infections were detected in only one of three species of *Crematogaster*, i.e., *C. vagula* Wheeler (26.67%, 6 sites) (Table 2). These results showed that *Lactobacillus* was more prevalent in Formicines (especially in *Formica* ants) than in Myrmicinae ants.

**TABLE 2:** Average infection rates of *Lactobacillus* among 15 honeydew-feeding ants.

Subfamily	Ant species	Number of workers	Number of infected workers	Average infection rates (%)
Formicinae	*Formica japonica* Motschoulsky	90	76	84.4
	*Formica glauca* Ruzsky	90	56	62.2
	*Formica cunicularia* Latreille	110	80	72.7
	*Formica gagatoides* Ruzsky	10	8	80.0
	*Formica sanguinea* Latreille	10	9	90.0
	*Formica polyctena* Foerster	10	10	100.0
	*Lasius niger* (Linnaeus)	140	46	32.9
	*Lasius fuliginosus* (Latreille)	10	10	100.0
	*Camponotus japonicus* Mayr	70	30	42.9
	*Camponotus obscuripes* Mayr	10	6	60.0
Myrmicinae	*Pristomyrmex pungens* Mayr	10	10	100.0
	*Crematogaster vagula* Wheeler	60	16	26.7
	*Crematogaster bandarensis* Forel	10	0	0
	*Crematogaster artifex* Mayr	10	0	0
Dolichoderinae	*Dolichoderus sibiricus* Emery	10	0	0

### Isolation and Identification of Lactic Acid Bacteria Strains From the Digestive Tracts of Honeydew-Feeding Ants

Workers collected from seven species of honeydew-feeding ants (Supplementary Table 2) were vivisected to isolate lactic acid bacteria strains. Most lactic acid bacteria strains were isolated from the IBPs, crops of *F. cunicularia*, *F. fusca* Linnaeus and *F. japonica*, and whole individual workers of *C. vagula* and *Dolichoderus sibiricus* Emery. Few strains were isolated from two other ant species (*L. niger* and *P. pungens*) (Supplementary Table 2). In addition, no lactic acid bacteria strain was isolated from the midguts and hindguts of any ant species after 72 h incubation on MRS agar medium.

Isolated lactic acid bacterial strains formed milk white, smooth round, and concave colonies approximately 1–2 mm in diameter. However, some bacterial colonies were very small (approximately 0.1 mm in diameter). A total of 15 representative bacterial strains were selected and subjected to DNA-based molecular identification (Table 3). Molecular identification of these isolates displayed a similarity of 98–100% with the closest matching sequences in the NCBI database (Table 3), and *Lactobacillus sanfranciscensis* and *Weissella cibaria* were predominant bacterial isolates.

**TABLE 3:** Isolated lactic acid bacterial strains from honeydew-feeding ants.

Isolated strain	Most closely related bacteria	Sequence similarity	Accession number	Ant species	Tissue source	Tree species
La1	*Lactobacillus lindneri* (LC097078.1)	99.9%	MZ723326	*Formica cunicularia*	Crops	*Malus halliana*
La2	*Lactobacillus sanfranciscensis* (LC483557.7)	99.2%	MZ723327		Crops	*Cedrus deodara*
La3	*Weissella cibaria* (MG669650.1)	99. 6%	MZ723328		IBPs	*Cedrus deodara*
La4	*Lactobacillus sanfranciscensis* (LC483557.3)	99.2%	MZ723329		Crops	*Koelreuteria paniculata*
La5	*Lactobacillus sanfranciscensis* (LC483557.4)	99.1%	MZ723330		IBPs	*Koelreuteria paniculata*
La6	*Lactobacillus sanfranciscensis* (LC483557.8)	98.9%	MZ723331		Crops	*Aesculus chinensis*
La7	*Lactobacillus lindneri* (LC097078.3)	99.7%	MZ723332		Crops	*Aesculus chinensis*
La8	*Weissella cibaria* (MG669650.5)/*Weissella halotolerans* (LC064886.1)	99.5%	MZ723333		Crops	*Aesculus chinensis*
La9	*Fructobacillus* sp. (MH236788.1)	100%	MZ723334		Crops	*Aesculus chinensis*
La10	*Lactobacillus sanfranciscensis* (LC483557.1)	99.2%	MZ723335	*Formica fusca*	IBPs	*Sophora japonica*
La11	*Lactobacillus lindneri* (LC097078.2)	99.4%	MZ723336		Crops	*Sophora japonica*
La12	*Weissella cibaria* (MG669650.2)	99.9%	MZ723337	*Formica japonica*	Crops	*Cedrus deodara*
La13	*Weissella cibaria* (MG669650.4)	99.9%	MZ723338		IBPs	*Malus halliana*
La14	*Lactobacillus sanfranciscensis* (LC483557.6)	99.3%	MZ723339	*Lasius niger*	Heads	*Cedrus deodara*
La15	*Lactobacillus sanfranciscensis* (LC483557.3)	99.3%	MZ723340	*Crematogaster vagula*	Heads	*Albizzia julibrissin*

Maximum-likelihood phylogenetic analysis of the 16S rRNA genes of lactic acid bacteria isolated from ants in this study and from other sources showed that the bacteria comprised three different groups. In detail, one strain (La9) was clustered with *Fructobacillus* sp., which was most closely related to sequences from grains and fruits (Figure 4). Four strains (La3, La8, La12, and La13) were clustered tightly with *Weissella* sp., which had previously been isolated from fermented food (Figure 4). Ten strains (La1, La2, La4, La5, La6, La7, La10, La11, La14, and La15) were classified into *Lactobacillus* sp., which consisted of sequences from diverse habitats, including stinky tofu brine, wheat flour, and *Iridomyrmex* ants (Figure 4).

### Sugar Catabolism Ability of Isolated Lactic Acid Bacterial Strains

**FIGURE 5:**
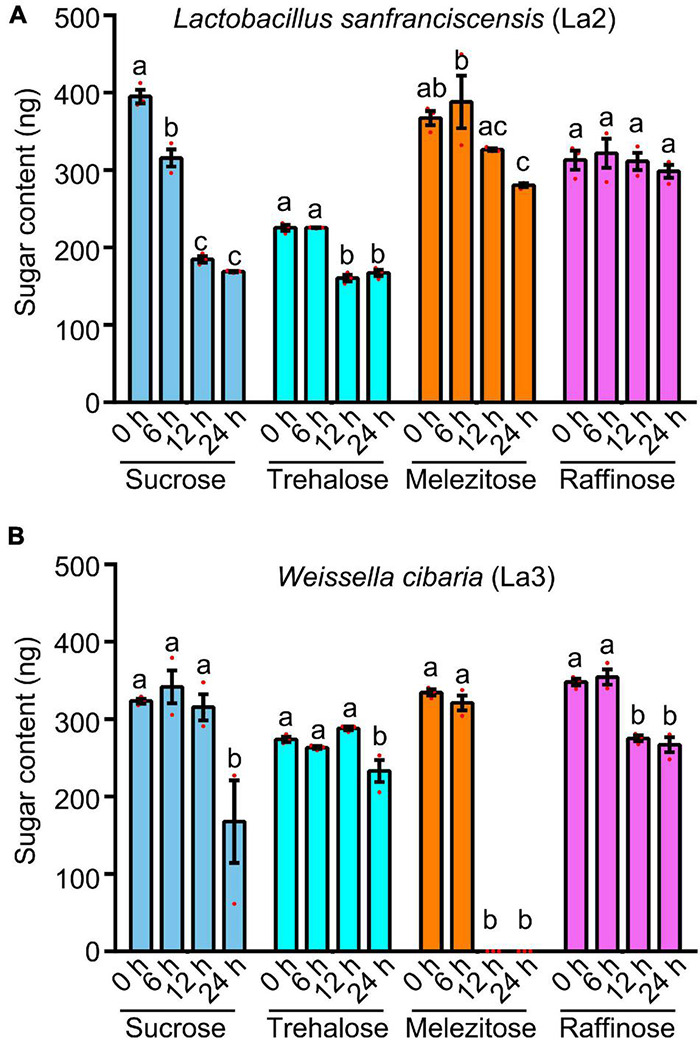
Catabolism of sugars (sucrose, trehalose, melezitose, and raffinose) by two strains of lactic acid bacteria. **(A)** *Lactobacillus sanfranciscensis* (La2). **(B)** *Weissella cibaria* (La3). Different letters indicate significant differences among different lines (Duncan’s multiple range test). Values are means ± SEM; *n* = 3.

Two predominant lactic acid bacterial isolates, i.e., *Lactobacillus sanfranciscensis* (La2) and *Weissella cibaria* (La3), were chosen to test their capacity for catabolizing four sugars (sucrose, trehalose, melezitose and raffinose). With the HPLC analysis, *Lactobacillus sanfranciscensis* (La2) can catabolize sucrose, trehalose, and melezitose (Figure 5A and Supplementary Figures 2A–F). *Weissella cibaria* (La3) can catabolize sucrose, melezitose, and raffinose (Figure 5B and Supplementary Figures 3A–F). These results revealed that lactic acid bacteria facilitate ants in utilizing sugars contained in honeydew.

## Discussion

This is the first study to investigate the bacterial communities of ants across different species and several colonies, especially in the context of ant-aphid mutualism. We aimed to identify the factors that shape the variations in the bacterial communities and their functions. Several studies on the bacterial composition of ants had previously reported that the factors influencing the bacterial communities are diverse, including diet, geography, species and phylogeny (Russell et al., 2009; Anderson et al., 2012; Hu et al., 2014, 2017; Sanders et al., 2014). Our results showed that several factors had obvious influence on the bacterial communities of formicines, such as trophic levels, phylogeny, colonies, and structures of digestive tract. We also found two main bacteria associated with the diet of formicines—*Lactobacillus* and uncultured Acetobacteraceae—that may facilitate ants in catabolizing honeydew. In addition, *Wolbachia* was a dominant bacterial genus widespread in most samples; although its function in ants is unclear, we suggest that it is transmitted vertically.

### The Diversity and Variation of Bacterial Communities in *Formica* and *Lasius* Ants

The three *Formica* ant species studied here mainly harbored *Wolbachia* and *Lactobacillus*, whereas *L. niger* was dominated by *Lactobacillus*, uncultured Acetobacteraceae, *Rickettsiella*, *Acinetobacter*, and *Escherichia*-*Shigella*. *Formica* ants and *L. niger* harbor different bacterial communities and occupy different trophic levels compared to the ants studied in previous research (Russell et al., 2009). For instance, predatory ants (Ecitoninae, Dorylinae, Aenictinae and Ponerinae) at a high trophic level possess Entomoplasmatales (*Mesoplasma* and *Spiroplasma*) and *Wolbachia* (Funaro et al., 2011; Zheng et al., 2021). Functionally herbivorous ants (Formicinae and Myrmicinae) at a low trophic level house *Blochmannia*, Rhizobiales, Pseudomonadales, Xanthomonadales, Opitutals, Flavobacteriales, and Burkholderiales (Russell et al., 2009; Anderson et al., 2012; Poulsen and Sapountzis, 2012). Predatory ants mainly prey on a N-rich diet and primarily harbor bacteria associated with their lifestyle. However, herbivorous ants feed on carbohydrate-rich, N-poor liquids such as extrafloral nectar, plant wound secretions, and insect honeydew (Hölldobler and Wilson, 1990; Blüthgen et al., 2000; Blüthgen and Fiedler, 2002; Davidson et al., 2004). Therefore, many such species harbor bacteria that could provide valuable nutritional contributions (van Borm et al., 2002a; Hu et al., 2018). Bacteria may therefore facilitate convergent evolution of herbivory across ants, and are considered to be a major force in ant evolution (Russell et al., 2009).

Alongside the differences between ants at different trophic level, closely related ants also harbor similar microbiota compared to that in distantly related species from similar trophic niches (Currie et al., 2003; Anderson et al., 2012; Sanders et al., 2014). Our results showed that the digestive tracts of the three *Formica* ant species shared dominant bacterial communities (*Wolbachia* and *Lactobacillus*), but showed differences with the bacterial communities of *L. niger* (*Lactobacillus*, uncultured Acetobacteraceae, *Rickettsiella*, *Acinetobacter*, and *Escherichia*-*Shigella*). NMDS plots revealed the same trend that no difference was observed in bacterial communities between the three *Formica* ant species, whereas the difference was obvious between *Formica* ants and *L. niger*. Although the *Formica* ants and *L. niger* studied here all feed on honeydew, their bacterial communities were different, which implied that phylogeny has a greater effect on bacterial communities than diet when ants occupied the same trophic level.

In addition to variations in bacterial communities across ant species, our results showed colony-level signatures of bacterial communities within ant species. Similar trends have been reported in *Pheidole*, *Polyrhachis*, and *Temnothorax* (Ramalho et al., 2017; Martins and Moreau, 2020; Ronque et al., 2020). Ants are social insects that live in populous colonies, and individuals in the same colony share the same space and food (Hölldobler and Wilson, 1990), which favor the exchange of bacteria among nestmates (Archie and Theis, 2011). Differences in bacterial communities between colonies of the same ant species can be explained by host genetic variability, which would lead to a colony-level natural selection on bacteria (Hu et al., 2014). Geographical isolation may lead to the lack of social communication between colonies, which can increase the discrepancy of bacterial communities between colonies. In addition, bacterial communities are related to group-specific odors, suggesting that the members of the same social group harbor similar odor-producing bacterial communities, which is potentially important for within-group recognition (Teseo et al., 2019).

On analyzing the bacterial communities in different digestive tract regions of workers and broods, we found that the bacterial communities in the IBPs and crops were different from those in the larvae, pupae and cocoons. However, bacterial communities in the midguts and hindguts showed overlap with those from pupae and larvae. This is possibly due to the difference in diet between workers and broods. Workers are the major recipients of honeydew, whereas broods are provided with protein (Lange, 1960). Therefore, workers harbor *Lactobacillus* retained in the IBPs and crops that can help them catabolize honeydew. The bacterial communities in the midguts, hindguts and broods were largely infected with *Wolbachia*, leading to the similarity of bacterial communities in these samples.

### Structural Variation Impacts Bacterial Communities in Different Digestive Regions

*Lactobacillus* was stable and dominant in the IBPs and crops of the three *Formica* species, but seldom presented in the midguts and hindguts. The NMDS plot also showed similarity in the bacterial communities between the IBPs and crops, which were different from those of the midguts and hindguts. This pattern is closely related to the function and structure of the digestive tract. In formicines, workers mainly feed on liquid food and rely on the IBPs to filter solid food. The flow of liquid food allows the microbe to pass through the digestive tract and be retained temporarily in the crop, and probably leading to the similarity in bacterial communities in the IBPs and crops. However, a previous study on bacterial communities in *O. monticola* and *E. javanus* has shown opposite results, in that the IBPs and crops exhibited different bacterial communities (Zheng et al., 2021). This is mainly due to the structural and functional differences of the IBPs between formicines and prey-feeding ants: the IBP is well-developed in prey-feeding ants (Edwards, 1980) and is more capable of obstructing solid particles and bacteria than in formicines, and thus leading to the differences in bacterial communities in the IBPs and crops of predatory ants. In addition, we speculate that the IBPs have some function in digesting food and thus provide a suitable internal environment for *Lactobacillus*, which may help in fermenting foods rich in carbohydrates, e.g., honeydew.

However, bacterial communities in the midguts and hindguts were different from those in the IBPs and crops. Similar results have been observed in *Cephalotes* ants, in which Rhizobiales were dominant in the crop and proventriculus, but *Opitutus* was dominant in the midgut and hindgut (Lanan et al., 2016). This is mainly related to the structure of the proventriculus. The proventriculus is a part of the foregut, and is more than a simple connection between the crop and the midgut. The proventriculus is also responsible for obstructing the passage of large solid particles into the crop, and allows liquid food transfer to the midgut. With the evolution of ants, the structure of the proventriculus represents a large degree of diversification among Formicidae (Caetano, 1990). The proventriculus is highly elaborated in Formicinae, Pseudomyrmicinae and Dolichoderinae, but not elaborated in primitive Ponerinae. The elaborated proventriculus obstructs not only solid particles but also bacteria harbored in the food flow, resulting in the difference in bacterial communities between the crops and midguts. However, in Ponerinae, the proventriculus is not elaborated and cannot obstruct bacteria flow backward with the food, leading to the similarity of bacterial communities in the crops and midguts, this can be verified in our previous study on two ponerine ants (Zheng et al., 2021).

### Bacteria Associated With Sugar Catabolism

In this study, *Lactobacillus lindneri* (La1, La7, and La11), *Lactobacillus sanfranciscensis* (La2, La4, La5, La6, La10, La14, and La15), *Weissella cibaria* (La3, La8, La12, and La13) and *Fructobacillus* sp. (La9) were isolated from honeydew-feeding ants using culture-dependent methods, and they were dominant in the IBPs and crops of *Formica* ants. Previous studies also identified *Lactobacillus* in several ants, including *Brachymymex*, *Linepithema humile* (Mayr), *F. exsecta* Nylander, *Polyrhachis*, and *Solenopsis invicta* Buren (Ishak et al., 2011; Johansson et al., 2013; Hu et al., 2017; Ramalho et al., 2017; Ivens et al., 2018). *Fructobacillus* was the predominant bacteria in the IBPs of *C. japonicus* (Zhang et al., 2018), and also presented in the crops, midguts and hindguts of *C. japonicus* (He et al., 2011; Li et al., 2012).

Ants and bees prefer sugar-rich foods, e.g., honeydew, nectar and pollen. Besides being identified in ants, lactic acid bacteria are more common in honey bees and bumble bees. A previous study on *Apis mellifera* (Linnaeus) showed that carbohydrate-degrading enzymes (pectin degradation enzyme, glycoside hydrolase and polysaccharide hydrolase) can be found in *Lactobacillus*, *Bifidobacterium*, and *Gilliamella apicola* (Engel et al., 2012). Analysis of sugar catabolism in our present study also showed that two predominant isolated lactic acid bacterial strains [*Lactobacillus sanfranciscensis* (La2) and *Weissella cibaria* (La3)] could catabolize different sugars (sucrose, melezitose, trehalose, and raffinose) contained in honeydew, indicating that lactic acid bacteria potentially may play an important role for ants nourishing from carbohydrate-rich foods in nature, although their importance for ant fitness awaits further investigation. In addition to catabolizing sugar-rich food, lactic acid bacteria also express antibacterial properties by secreting lactic acid to create acidize environmental conditions that other bacteria and fungi cannot tolerate to protect *Mycocepurus smithii* (Forel) (Kellner et al., 2015).

Lactic acid bacteria are known to be widely distributed in ants and perform vital functions for their hosts; however, how these bacteria are transmitted to ants remains unknown. There exist at least two possible transmission routes of lactic acid bacteria: (i) the bacteria are acquired transiently from aphids, secreted honeydew or plants, or (ii) the bacteria are vertically transmitted and persistently colonize the digestive tract. A previous study detected seven bacteria (*Buchnera*, *Serratia*, *Regiella*, *Hamiltonella*, *Rickettsia*, *Spiroplasma* and *Arsenophonus*) from various aphid species (Oliver et al., 2010), but no lactic acid bacteria were detected. This implis that ants cannot acquire lactic acid bacteria from aphids. Coincidentally, a *Lactobacillus plantarum* strain had been previously isolated from the honeydew produced by *Coccus hesperidum* (Linnaeus) (Gustaw et al., 2018), and it is likely that ants gained lactic acid bacteria from sugar-rich foods. In addition, plants and insect herbivores formed intricate and diverse relationships with microbes, and plant-associated microbial communities are more diverse than insect herbivores (Sugio et al., 2015). A recent study showed that 21 species of lactic acid bacteria belonging to the genera *Enterococcus*, *Fructobacillus*, *Lactobacillus*, *Lactococcus*, *Leuconostoc* and *Weissella* were isolated from fruits and flowers which are food sources for insect herbivores (Ruiz Rodríguez et al., 2019), indicating that lactic acid bacteria should have a plant-related origin for insect herbivores. Moreover, lactic acid bacteria were seldom present in the larvae, pupae and cocoons of these ants, indicating that the latter conjecture may be ruled out. However, more studies should be conducted to confirm the possible transmission routes of lactic acid bacteria in the future.

With the evolution of Hymenoptera and their associated lactic acid bacteria, honey and bumble bees have formed host specificity with the Firm3 and Firm4 clades of *Lactobacillus* (McFrederick et al., 2013). Since ants and other social insects of Hymenoptera have not been found to exhibit host specificity with associated lactic acid bacteria, the specificity between lactic acid bacteria and Hymenopteran hosts is considered to be the exception rather than the rule (McFrederick et al., 2013). This is the first report of rich lactic acid bacteria distributed in the IBPs and crops of ants preferring honeydew, suggesting that these bacteria may be tightly associated with honeydew-feeding ants and have a potential function in the utilization of honeydew resources in nature. Our research provides interesting clues regarding the relationship between ants, hemipteran and microbes.

Acetobacteraceae—a family of acetic acid-producing bacteria that thrive in sugar-rich environments—was identified in the crops of *Formica* ants and heads and gasters of *L. niger*. Unfortunately, the bacteria could not be assigned into genus or species level even though we sequenced almost the full-length of their 16S rRNA genes. Acetobacteraceae has been previously found in ants with carbohydrate-rich diets, including honeydew-feeding ants (*Lasius*), wood ants (*Formica*), carpenter ants (*Camponotus*), and Argentine ants (*Linepithema*) (Russell et al., 2009; Brown and Wernegreen, 2016; Hu et al., 2017; Ivens et al., 2018). Dominant *Acetobacter aceti* has been detected in the crop of lab-raised *Camponotus fragilis* workers but not in other gut sections and filed colonies, and it has been suggested that the bacteria originated from the honey diet (He et al., 2014). Two Acetobacteraceae OTUs were found to be abundant and widespread in *Camponotus chromaiodes* colonies, and they were considered to be helpful in digesting the ants’ sugary honeydew and have built symbiotic relationship with *Camponotus* ants (Brown and Wernegreen, 2016). More studies should be conducted to elucidate the roles of Acetobacteraceae in honeydew-feeding ants.

### The Dominance of *Wolbachia*

*Wolbachia* was dominant in *Formica* ants in this study, and its relative abundance exceeded 50% in the midguts and hindguts of workers, larvae, and pupae. *Wolbachia* has been estimated to infect 34% of ant species (Russell, 2012), which is mainly localized in the germline and various somatic tissues of workers and queens (Andersen et al., 2012; Frost et al., 2014; Zhukova et al., 2017; Ramalho et al., 2018; Zheng et al., 2021). Based on our present results, we hypothesize that *Wolbachia* is transmitted vertically in *Formica* ants and restricted in the midguts and hindguts. Since *Wolbachia* is seldom presented in the IBPs and crops, it could not have been transmitted from the environment via food to the midguts and hindguts. The potential route of vertical transmission of *Wolbachia* is that it infected entire colony through a single infected queen, and then transmitted vertically from queens to eggs, which has been reported in several ant species (Bouwma and Shoemaker, 2011; Ramalho et al., 2018).

*Wolbachia* is known to manipulate the reproduction of solitary host to promote its own transmission through the induction of cytoplasmic incompatibility, parthenogenesis, and male-killing or feminization (Werren et al., 2008). There is some evidence to suggest that *Wolbachia* may influence sex ratios in social ants, but this has only been documented in *Monomorium pharaonic* (Linnaeus) (Singh and Linksvayer, 2020). Apart from their effects on reproduction, *Wolbachia* also appears to accelerate the colony life cycle of *M. pharaonic* and provide vitamin B for *Tapinoma melanocephalum* (Fabricius) (Cheng et al., 2019; Singh and Linksvayer, 2020). However, the effects of *Wolbachia* on *Formica* and other ants are largely unknown and merit further investigation. The difficulties in understanding whether *Wolbachia* has any impact on their host ants are due to two factors: first, most ants in a colony are non-breeding workers, if their reproduction was manipulated by *Wolbachia*, it would lead to an evolutionary impasse; and second, older workers may lose their infection or at least have *Wolbachia* in low enough titers to which are difficult to be detected.

## Data Availability Statement

The datasets generated in this study can be found in the NCBI SRA database under accession number PRJNA752991 and NCBI GenBank under accession numbers MZ723326–MZ723340.

## Author Contributions

HH and ZhoZ designed the research. ZhoZ, XH, ZhiZ, and MZ performed the research. ZhoZ, MZ, and YX analyzed the data. ZhoZ, CW, and HH wrote the manuscript. All authors contributed to the article and approved the submitted version.

## Conflict of Interest

The authors declare that the research was conducted in the absence of any commercial or financial relationships that could be construed as a potential conflict of interest.

## Publisher’s Note

All claims expressed in this article are solely those of the authors and do not necessarily represent those of their affiliated organizations, or those of the publisher, the editors and the reviewers. Any product that may be evaluated in this article, or claim that may be made by its manufacturer, is not guaranteed or endorsed by the publisher.

## Footnotes

1
https://www.microbiomeanalyst.ca/MicrobiomeAnalyst/home.xhtml

## References

[B1] Anahtar M. N. Bowman B. A. Kwon D. S. (2016). Efficient nucleic acid extraction and 16S rRNA gene sequencing for bacterial community characterization. *J. Vis. Exp.* 110:e53939. 10.3791/53939 27168460 PMC4941931

[B2] Andersen S. B. Boye M. Nash D. R. Boomsma J. J. (2012). Dynamic *Wolbachia* prevalence in *Acromyrmex* leaf-cutting ants: potential for a nutritional symbiosis. *J. Evol. Biol.* 25 1340–1350. 10.1111/j.1420-9101.2012.02521.x 22530696

[B3] Anderson K. E. Russell J. A. Moreau C. S. Kautz S. Sullam K. E. Hu Y. (2012). Highly similar microbial communities are shared among related and trophically similar ant species. *Mol. Ecol.* 21 2282–2296. 10.1111/j.1365-294X.2011.05464.x 22276952

[B4] Archie E. A. Theis K. R. (2011). Animal behaviour meets microbial ecology. *Anim. Behav.* 82 425–436. 10.1016/j.anbehav.2011.05.029

[B5] Billen J. Buschinger A. (2000). Morphology and ultrastructure of a specialized bacterial pouch in the digestive tract of *Tetraponera* ants (Formicidae, Pseudomyrmecinae). *Arthropod. Struct. Dev.* 29 259–266. 10.1016/s1467-8039(00)00029-3 18088931

[B6] Blüthgen N. Fiedler K. (2002). Interactions between weaver ants *Oecophylla smaragdina*, homopterans, trees and lianas in an Australian rain forest canopy. *J. Anim. Ecol.* 71 793–801. 10.1046/j.1365-2656.2002.00647.x

[B7] Blüthgen N. Verhaagh M. Goitía W. Jaffé K. Morawetz W. Barthlott W. (2000). How plants shape the ant community in the Amazonian rainforest canopy: the key role of extrafloral nectaries and homopteran honeydew. *Oecologia* 125 229–240. 10.1007/s004420000449 24595834

[B8] Bolger A. M. Lohse M. Usadel B. (2014). Trimmomatic: a flexible trimmer for Illumina sequence data. *Bioinformatics* 30 2114–2120. 10.1093/bioinformatics/btu170 24695404 PMC4103590

[B9] Bolyen E. Rideout J. R. Dillon M. R. Bokulich N. Abnet C. C. Al-Ghalith G. A. (2019). Reproducible, interactive, scalable and extensible microbiome data science using QIIME 2. *Nat. Biotechnol.* 37 852–857. 10.1038/s41587-019-0209-9 31341288 PMC7015180

[B10] Bouwma A. M. Shoemaker D. (2011). *Wolbachia* wSinvictaA infections in natural populations of the fire ant *Solenopsis invicta*: testing for phenotypic effects. *J. Insect Sci.* 11:11. 10.1673/031.011.0111 21526927 PMC3281330

[B11] Brady S. G. Schultz T. R. Fisher B. L. Ward P. S. (2006). Evaluating alternative hypotheses for the early evolution and diversification of ants. *Proc. Natl. Acad. Sci. U.S.A.* 103 18172–18177. 10.1073/pnas.0605858103 17079492 PMC1838725

[B12] Brown B. P. Wernegreen J. J. (2016). Deep divergence and rapid evolutionary rates in gut-associated Acetobacteraceae of ants. *BMC Microbiol.* 16:140. 10.1186/s12866-016-0721-8 27400652 PMC4939635

[B13] Bution M. L. Caetano F. H. (2008). Ileum of the *Cephalotes* ants: a specialized structure to harbor symbionts microorganisms. *Micron* 39 897–909. 10.1016/j.micron.2007.11.008 18187330

[B14] Caetano F. (1990). “Morphology of the digestive tract and associated excretory organs of ants,” in *Applied Myrmecology—A World Perspective*, eds Vander Meer R. K. Jaffé K. Cedeno A. (Boulder, CO: Westview Press), 119–129.

[B15] Caetano F. da Cruz-Landim C. (1985). Presence of microorganisms in the alimentary canal of ants of the tribe Cephalotini (Myrmicinae): location and relationship with intestinal structures. *Naturalia (São José do Rio Preto)* 10 37–47.

[B16] Caetano F. Jaffe K. Crewe R. (1994). “The digestive tract of *Cataulacus* ants: presence of microorganisms in the ileum,” in *Les Insectes Sociaux*, eds Lenoir A. Arnold G. Lepage M. (Paris: Université Paris Nord), 391.

[B17] Callahan B. J. McMurdie P. J. Holmes S. P. (2017). Exact sequence variants should replace operational taxonomic units in marker-gene data analysis. *ISME J.* 11 2639–2643. 10.1038/ismej.2017.119 28731476 PMC5702726

[B18] Callahan B. J. McMurdie P. J. Rosen M. J. Han A. W. Johnson A. J. A. Holmes S. P. (2016). DADA2: high-resolution sample inference from Illumina amplicon data. *Nat. Methods* 13 581–583. 10.1038/nmeth.3869 27214047 PMC4927377

[B19] Cheng D. Chen S. Huang Y. Pierce N. E. Riegler M. Yang F. (2019). Symbiotic microbiota may reflect host adaptation by resident to invasive ant species. *PLoS Path.* 15:e1007942. 10.1371/journal.ppat.1007942 31323076 PMC6668852

[B20] Currie C. R. Scott J. A. Summerbell R. C. Malloch D. (1999). Fungus-growing ants use antibiotic-producing bacteria to control garden parasites. *Nature* 398 701–704. 10.1038/19519

[B21] Currie C. R. Wong B. Stuart A. E. Schultz T. R. Rehner S. A. Mueller U. G. (2003). Ancient tripartite coevolution in the attine ant-microbe symbiosis. *Science* 299 386–388. 10.1126/science.1078155 12532015

[B22] Davidson D. W. Cook S. C. Snelling R. R. (2004). Liquid-feeding performances of ants (Formicidae): ecological and evolutionary implications. *Oecologia* 139 255–266. 10.1007/s00442-004-1508-4 15034777

[B23] Davis N. M. Proctor D. M. Holmes S. P. Relman D. A. Callahan B. J. (2018). Simple statistical identification and removal of contaminant sequences in marker-gene and metagenomics data. *Microbiome* 6:226. 10.1186/s40168-018-0605-2 30558668 PMC6298009

[B24] de Oliveira T. B. Ferro M. Bacci M. de Souza D. J. Fontana R. Delabie J. H. C. (2016). Bacterial communities in the midgut of ponerine ants (Hymenoptera: Formicidae: Ponerinae). *Sociobiology* 63 637–644. 10.13102/sociobiology.v63i1.882

[B25] Degnan P. H. Lazarus A. B. Wernegreen J. J. (2005). Genome sequence of *Blochmannia pennsylvanicus* indicates parallel evolutionary trends among bacterial mutualists of insects. *Genome Res.* 15 1023–1033. 10.1101/gr.3771305 16077009 PMC1182215

[B26] Dhariwal A. Chong J. Habib S. King I. L. Agellon L. B. Xia J. G. (2017). MicrobiomeAnalyst: a web-based tool for comprehensive statistical, visual and meta-analysis of microbiome data. *Nucleic Acids Res.* 45 W180–W188. 10.1093/nar/gkx295 28449106 PMC5570177

[B27] Douglas A. (1993). The nutritional quality of phloem sap utilized by natural aphid populations. *Ecol. Entomol.* 18 31–38. 10.1111/j.1365-2311.1993.tb01076.x

[B28] Edwards R. (1980). *Social WASPS. Their Biology and Control.* Crawley: Rentokil Ltd.

[B29] Eisner T. (1957). A comparative morphological study of the proventriculus of ants (Hymenoptera: Formicidae). *Bull. Mus. Comp. Zool.* 116 439–490.

[B30] Eisner T. Happ G. (1962). The infrabuccal pocket of a formicine ant: a social filtration device. *Psyche* 69 107–116.

[B31] Engel P. Martinson V. G. Moran N. A. (2012). Functional diversity within the simple gut microbiota of the honey bee. *Proc. Natl. Acad. Sci. U.S.A..* 109 11002–11007. 10.1073/pnas.1202970109 22711827 PMC3390884

[B32] Fischer M. K. Völkl W. Schopf R. Hoffmann K. H. (2002). Age-specific patterns in honeydew production and honeydew composition in the aphid *Metopeurum fuscoviride*: implications for ant-attendance. *J. Insect Physiol.* 48 319–326. 10.1016/s0022-1910(01)00179-2 12770106

[B33] Frank J. A. Reich C. I. Sharma S. Weisbaum J. S. Wilson B. A. Olsen G. J. (2008). Critical evaluation of two primers commonly used for amplification of bacterial 16S rRNA genes. *Appl. Environ. Microbiol.* 74 2461–2470. 10.1128/AEM.02272-07 18296538 PMC2293150

[B34] Frost C. L. Pollock S. W. Smith J. E. Hughes W. O. H. (2014). *Wolbachia* in the flesh: symbiont intensities in germ-line and somatic tissues challenge the conventional view of *Wolbachia* transmission routes. *PLoS One* 9:e95122. 10.1371/journal.pone.0095122 24988478 PMC4079706

[B35] Funaro C. F. Kronauer D. J. C. Moreau C. S. Goldman-Huertas B. Pierce N. E. Russell J. A. (2011). Army ants harbor a host-specific clade of Entomoplasmatales bacteria. *Appl. Environ. Microbiol.* 77 346–350. 10.1128/aem.01896-10 21075876 PMC3019723

[B36] Gaudermann P. Vogl I. Zientz E. Silva F. J. Moya A. Gross R. (2006). Analysis of and function predictions for previously conserved hypothetical or putative proteins in *Blochmannia floridanus*. *BMC Microbiol.* 6:1. 10.1186/1471-2180-6-1 16401340 PMC1360075

[B37] Gotwald W. H. (1969). *Comparative Morphological Studies of the Ants, with Particular Reference to the Mouthparts (Hymenoptera: Formicidae).* Ithaca, N.Y: Agricultural Experiment Station.

[B38] Gustaw K. Michalak M. Polak-Berecka M. Wasko A. (2018). Isolation and characterization of a new fructophilic *Lactobacillus plantarum* FPL strain from honeydew. *Ann. Microbiol.* 68 459–470. 10.1007/s13213-018-1350-2 29983672 PMC6008367

[B39] He H. Chen Y. Y. Zhang Y. L. Wei C. (2011). Bacteria associated with gut lumen of *Camponotus japonicus* Mayr. *Environ. Entomol.* 40 1405–1409. 10.1603/en11157 22217755

[B40] He H. Wei C. Wheeler D. E. (2014). The gut bacterial communities associated with lab-raised and field-collected ants of *Camponotus fragilis* (Formicidae: Formicinae). *Curr. Microbiol.* 69 292–302. 10.1007/s00284-014-0586-8 24748441

[B41] Heilig H. Zoetendal E. G. Vaughan E. E. Marteau P. Akkermans A. D. L. de Vos W. M. (2002). Molecular diversity of *Lactobacillus* spp. and other lactic acid bacteria in the human intestine as determined by specific amplification of 16S ribosomal DNA. *Appl. Environ. Microbiol.* 68 114–123. 10.1128/aem.68.1.114-123.2002 11772617 PMC126540

[B42] Hölldobler B. Wilson E. O. (1990). *The Ants.* Cambridge, MA: Press of Harvard University.

[B43] Hu Y. Holway D. A. Lukasik P. Chau L. Kay A. D. LeBrun E. G. (2017). By their own devices: invasive Argentine ants have shifted diet without clear aid from symbiotic microbes. *Mol. Ecol.* 26 1608–1630. 10.1111/mec.13991 28026894

[B44] Hu Y. Lukasik P. Moreau C. S. Russell J. A. (2014). Correlates of gut community composition across an ant species (*Cephalotes varians*) elucidate causes and consequences of symbiotic variability. *Mol. Ecol.* 23 1284–1300. 10.1111/mec.12607 24286170

[B45] Hu Y. Sanders J. G. Lukasik P. D’Amelio C. L. Millar J. S. Vann D. R. (2018). Herbivorous turtle ants obtain essential nutrients from a conserved nitrogen-recycling gut microbiome. *Nat. Commun.* 9:964. 10.1038/s41467-018-04935-w 29511180 PMC5840417

[B46] Hussain A. Forrest J. Dixon A. (1974). Sugar, organic-acid, phenolic acid and plant-growth regulator content of extracts of honeydew of the aphid *Myzus persicae* and of its host plant, *Raphanus sativus*. *Ann. Appl. Biol.* 78 65–73. 10.1111/j.1744-7348.1974.tb01486.x 19280790

[B47] Ishak H. D. Plowes R. Sen R. Kellner K. Meyer E. Estrada D. A. (2011). Bacterial diversity in *Solenopsis invicta* and *Solenopsis geminata* ant colonies characterized by 16S amplicon 454 pyrosequencing. *Microb. Ecol.* 61 821–831. 10.1007/s00248-010-9793-4 21243351

[B48] Ivens A. B. F. Gadau A. Kiers E. T. Kronauer D. J. C. (2018). Can social partnerships influence the microbiome? Insights from ant farmers and their trophobiont mutualists. *Mol. Ecol.* 27 1898–1914. 10.1111/mec.14506 29411455 PMC5935579

[B49] Johansson H. Dhaygude K. Lindström S. Helanterä H. Sundström L. Trontti K. (2013). A metatranscriptomic approach to the identification of microbiota associated with the ant *Formica exsecta*. *PLoS One* 8:e79777. 10.1371/journal.pone.0079777 24260298 PMC3832538

[B50] Kellner K. Ishak H. D. Linksvayer T. A. Mueller U. G. (2015). Bacterial community composition and diversity in an ancestral ant fungus symbiosis. *FEMS Microbiol. Ecol.* 91:fiv073. 10.1093/femsec/fiv073 26113689

[B51] Kumar S. Stecher G. Tamura K. (2016). MEGA7: molecular evolutionary genetics analysis version 7.0 for bigger datasets. *Mol. Biol. Evol.* 33 1870–1874. 10.1093/molbev/msw054 27004904 PMC8210823

[B52] Lanan M. C. Rodrigues P. A. P. Agellon A. Jansma P. Wheeler D. E. (2016). A bacterial filter protects and structures the gut microbiome of an insect. *ISME J.* 10 1866–1876. 10.1038/ismej.2015.264 26872040 PMC5029173

[B53] Lange R. (1960). Modellversuche über den nahrungsbedarf von völkern der kahlrückigen waldameise *Formica polyctena* Först. *Z. Angew. Entomol.* 46 200–208.

[B54] Leroy P. D. Wathelet B. Sabri A. Francis F. Verheggen F. J. Capella Q. (2011). Aphid-host plant interactions: does aphid honeydew exactly reflect the host plant amino acid composition? *Arthropod Plant Interact.* 5 193–199. 10.1007/s11829-011-9128-5

[B55] Li X. P. Nan X. N. Wei C. He H. (2012). The gut bacteria associated with *Camponotus japonicus* Mayr with culture-dependent and DGGE methods. *Curr. Microbiol.* 65 610–616. 10.1007/s00284-012-0197-1 22878556

[B56] Little A. E. F. Murakami T. Mueller U. G. Currie C. R. (2003). The infrabuccal pellet piles of fungus-growing ants. *Naturwissenschaften* 90 558–562. 10.1007/s00114-003-0480-x 14676952

[B57] Little A. E. F. Murakami T. Mueller U. G. Currie C. R. (2006). Defending against parasites: fungus-growing ants combine specialized behaviours and microbial symbionts to protect their fungus gardens. *Biol. Lett.* 2 12–16. 10.1098/rsbl.2005.0371 17148313 PMC1617182

[B58] Łukasik P. Newton J. A. Sanders J. G. Hu Y. Moreau C. S. Kronauer D. J. C. (2017). The structured diversity of specialized gut symbionts of the New World army ants. *Mol. Ecol.* 26 3808–3825. 10.1111/mec.14140 28393425

[B59] Magoč T. Salzberg S. L. (2011). FLASH: fast length adjustment of short reads to improve genome assemblies. *Bioinformatics* 27 2957–2963. 10.1093/bioinformatics/btr507 21903629 PMC3198573

[B60] Martins C. Moreau C. S. (2020). Influence of host phylogeny, geographical location and seed harvesting diet on the bacterial community of globally distributed *Pheidole* ants. *PeerJ* 8:e8492. 10.7717/peerj.8492 32117618 PMC7006521

[B61] McFrederick Q. S. Cannone J. J. Gutell R. R. Kellner K. Plowes R. M. Mueller U. G. (2013). Specificity between *Lactobacilli* and Hymenopteran hosts is the exception rather than the rule. *Appl. Environ. Microbiol.* 79 1803–1812. 10.1128/aem.03681-12 23291551 PMC3592248

[B62] Mittler T. (1958). Studies on the feeding and nutrition of *Tuberolachnus salignus* (Gmelin) (Homoptera, Aphididae): II. The nitrogen and sugar composition of ingested phloem sap and excreted honeydew. *J. Exp. Biol.* 35 74–84. 10.1242/jeb.35.1.74

[B63] Moreau C. S. Bell C. D. Vila R. Archibald S. B. Pierce N. E. (2006). Phylogeny of the ants: diversification in the age of angiosperms. *Science* 312 101–104. 10.1126/science.1124891 16601190

[B64] Nielsen D. S. Moller P. L. Rosenfeldt V. Paerregaard A. Michaelsen K. F. Jakobsen M. (2003). Case study of the distribution of mucosa-associated *Bifidobacterium* species, *Lactobacillus* species, and other lactic acid bacteria in the human colon. *Appl. Environ. Microbiol.* 69 7545–7548. 10.1128/aem.69.12.7545-7548.2003 14660412 PMC309914

[B65] Oliver K. M. Degnan P. H. Burke G. R. Moran N. A. (2010). Facultative symbionts in aphids and the horizontal transfer of ecologically important traits. *Annu. Rev. Entomol.* 55 247–266. 10.1146/annurev-ento-112408-085305 19728837

[B66] Poulsen M. Sapountzis P. (2012). Behind every great ant, there is a great gut. *Mol. Ecol.* 21 2054–2057. 10.1111/j.1365-294X.2012.05510.x 22509766

[B67] Quast C. Pruesse E. Yilmaz P. Gerken J. Schweer T. Yarza P. (2013). The SILVA ribosomal RNA gene database project: improved data processing and web-based tools. *Nucleic Acids Res.* 41 D590–D596. 10.1093/nar/gks1219 23193283 PMC3531112

[B68] Ramalho M. O. Bueno O. C. Moreau C. S. (2017). Microbial composition of spiny ants (Hymenoptera: Formicidae: *Polyrhachis*) across their geographic range. *BMC Evol. Biol.* 17:96. 10.1186/s12862-017-0945-8 28381207 PMC5382451

[B69] Ramalho M. O. Vieira A. S. Pereira M. C. Moreau C. S. Bueno O. C. (2018). Transovarian transmission of *Blochmannia* and *Wolbachia* endosymbionts in the neotropical weaver ant *Camponotus textor* (Hymenoptera, Formicidae). *Curr. Microbiol.* 75 866–873. 10.1007/s00284-018-1459-3 29468305

[B70] Richter A. Keller R. A. Rosumek F. B. Economo E. P. Garcia F. H. Beutel R. G. (2019). The cephalic anatomy of workers of the ant species *Wasmannia affinis* (Formicidae, Hymenoptera, Insecta) and its evolutionary implications. *Arthropod Struct. Dev.* 49 26–49. 10.1016/j.asd.2019.02.002 30738181

[B71] Ronque M. U. V. Lyra M. L. Migliorini G. H. Bacci M. Oliveira P. S. (2020). Symbiotic bacterial communities in rainforest fungus-farming ants: evidence for species and colony specificity. *Sci. Rep.* 10:10172. 10.1038/s41598-020-66772-6 32576863 PMC7311517

[B72] Ruiz Rodríguez L. G. Mohamed F. Bleckwedel J. Medina R. De Vuyst L. Hebert E. M. (2019). Diversity and functional properties of lactic acid bacteria isolated from wild fruits and flowers present in Northern Argentina. *Front. Microbiol.* 10:1091. 10.3389/fmicb.2019.01091 31164879 PMC6536596

[B73] Russell J. A. (2012). The ants (Hymenoptera: Formicidae) are unique and enigmatic hosts of prevalent *Wolbachia* (Alphaproteobacteria) symbionts. *Myrmecol. News* 16 7–23.

[B74] Russell J. A. Moreau C. S. Goldman-Huertas B. Fujiwara M. Lohman D. J. Pierce N. E. (2009). Bacterial gut symbionts are tightly linked with the evolution of herbivory in ants. *Proc. Natl. Acad. Sci. U.S.A.* 106 21236–21241. 10.1073/pnas.0907926106 19948964 PMC2785723

[B75] Sanders J. G. Powell S. Kronauer D. J. C. Vasconcelos H. L. Frederickson M. E. Pierce N. E. (2014). Stability and phylogenetic correlation in gut microbiota: lessons from ants and apes. *Mol. Ecol.* 23 1268–1283. 10.1111/mec.12611 24304129

[B76] Sauer C. Dudaczek D. Hölldobler B. Gross R. (2002). Tissue localization of the endosymbiotic bacterium “*Candidatus Blochmannia floridanus*” in adults and larvae of the carpenter ant *Camponotus floridanus*. *Appl. Environ. Microbiol.* 68 4187–4193. 10.1128/aem.68.9.4187-4193.2002 12200264 PMC124124

[B77] Schmieder R. Edwards R. (2011). Quality control and preprocessing of metagenomic datasets. *Bioinformatics* 27 863–864. 10.1093/bioinformatics/btr026 21278185 PMC3051327

[B78] Schröder D. Deppisch H. Obermayer M. Krohne G. Stackebrandt E. Hölldobler B. (1996). Intracellular endosymbiotic bacteria of *Camponotus* species (carpenter ants): systematics, evolution and ultrastructural characterization. *Mol. Microbiol.* 21 479–489. 10.1111/j.1365-2958.1996.tb02557.x 8866472

[B79] Segers F. Kaltenpoth M. Foitzik S. (2019). Abdominal microbial communities in ants depend on colony membership rather than caste and are linked to colony productivity. *Ecol. Evol.* 9 13450–13467. 10.1002/ece3.5801 31871657 PMC6912891

[B80] Singh R. Linksvayer T. A. (2020). *Wolbachia*-infected ant colonies have increased reproductive investment and an accelerated life cycle. *J. Exp. Biol.* 223:jeb220079. 10.1242/jeb.220079 32253286

[B81] Stadler B. Dixon A. F. G. (2005). Ecology and evolution of aphid-ant interactions. *Annu. Rev. Ecol. Evol. Syst.* 36 345–372. 10.1146/annurev.ecolsys.36.091704.175531

[B82] Sugio A. Dubreuil G. Giron D. Simon J. C. (2015). Plant–insect interactions under bacterial influence: ecological implications and underlying mechanisms. *J. Exp. Bot.* 66 467–478. 10.1093/jxb/eru435 25385767

[B83] Teseo S. van Zweden J. S. Pontieri L. Kooij P. W. Sorensen S. J. Wenseleers T. (2019). The scent of symbiosis: gut bacteria may affect social interactions in leaf-cutting ants. *Anim. Behav.* 150 239–254. 10.1016/j.anbehav.2018.12.017

[B84] Urbani C. B. De Andrade M. (1997). Pollen eating, storing, and spitting by ants. *Naturwissenschaften* 84 256–258. 10.1007/s001140050392

[B85] van Borm S. Billen J. Boomsma J. J. (2002a). The diversity of microorganisms associated with *Acromyrmex* leafcutter ants. *BMC Evol. Biol.* 2:9. 10.1186/1471-2148-2-9 12019020 PMC113273

[B86] van Borm S. Buschinger A. Boomsma J. J. Billen J. (2002b). Tetraponera ants have gut symbionts related to nitrogen-fixing root-nodule bacteria. *Proc. R. Soc. Lond. B Biol. Sci.* 269 2023–2027. 10.1098/rspb.2002.2101 12396501 PMC1691126

[B87] van Neerbos F. A. de Boer J. G. Salis L. Tollenaar W. Kos M. Vet L. E. (2020). Honeydew composition and its effect on life-history parameters of hyperparasitoids. *Ecol. Entomol.* 45 278–289. 10.1111/een.12799

[B88] Wang C. Billen J. Pan X. R. He H. (2018). Morphology and ultrastructure of the infrabuccal pocket and its lining epithelium in workers of *Ectomomyrmex javanus* (Hymenoptera: Formicidae). *Micron* 115 50–53. 10.1016/j.micron.2018.09.001 30199746

[B89] Wang C. Billen J. Wei C. He H. (2019). Morphology and ultrastructure of the infrabuccal pocket in *Camponotus japonicus* Mayr (Hymenoptera: Formicidae). *Insec. Soc.* 66 637–646. 10.1007/s00040-019-00726-8

[B90] Wang Y. C. Yu R. C. Yang H. Y. Chou C. C. (2003). Sugar and acid contents in soymilk fermented with lactic acid bacteria alone or simultaneously with bifidobacteria. *Food Microbiol.* 20 333–338. 10.1016/s0740-0020(02)00125-9

[B91] Way M. J. (1963). Mutualism between ants and honeydew-producing Homoptera. *Annu. Rev. Entomol.* 8 307–344. 10.1146/annurev.en.08.010163.001515

[B92] Werren J. H. Baldo L. Clark M. E. (2008). *Wolbachia*: master manipulators of invertebrate biology. *Nat. Rev. Microbiol.* 6 741–751. 10.1038/nrmicro1969 18794912

[B93] Zhang K. Wei C. Nan X. Wang Y. He H. (2018). Composition and diversity of microbes in the infrabuccal pocket of *Camponotus japonicus* (Hymenoptera: Formicidae). *Acta Entomol. Sin.* 61 686–697. 10.16380/j.kcxb.2018.06.007

[B94] Zheng Z. Hu X. Xu Y. Wei C. He H. (2021). Bacterial composition and civersity of the digestive tract of *Odontomachus monticola* Emery and *Ectomomyrmex javanus* Mayr. *Insects* 12:176. 10.3390/insects12020176 33671250 PMC7922086

[B95] Zhukova M. Sapountzis P. Schlott M. Boomsma J. J. (2017). Diversity and transmission of gut bacteria in *Atta* and *Acromyrmex* leaf-cutting ants during development. *Front. Microbiol.* 8:1942. 10.3389/fmicb.2017.01942 29067008 PMC5641371

