# Supplementary material for: Lactic Acid Bacteria Are Prevalent in the Infrabuccal Pockets and Crops of Ants That Prefer Aphid Honeydew
Source: Front Microbiol. 2022 Jan 21;12:785016. doi: 10.3389/fmicb.2021.785016 (PMC8814368; doi:10.3389/fmicb.2021.785016)
Supplement: Supplementary file 1 [file Data_Sheet_1.docx]

Supplementary Material

**
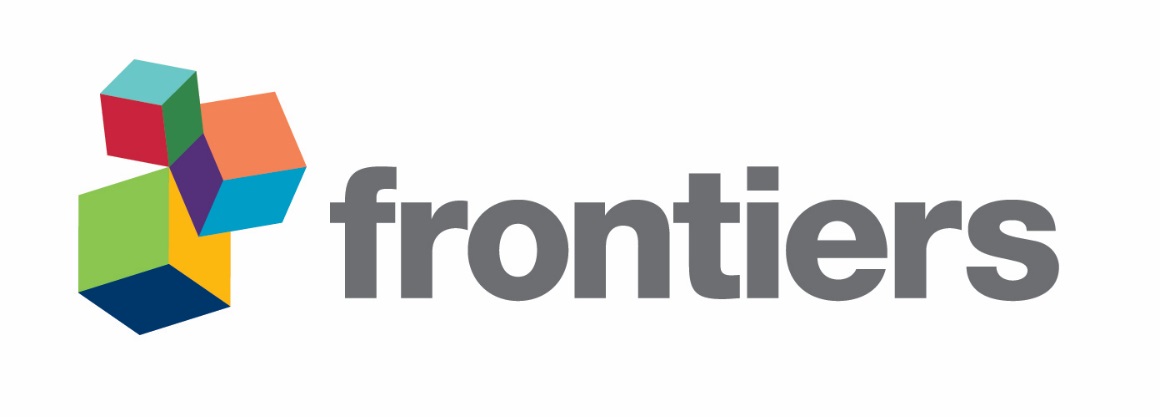
**


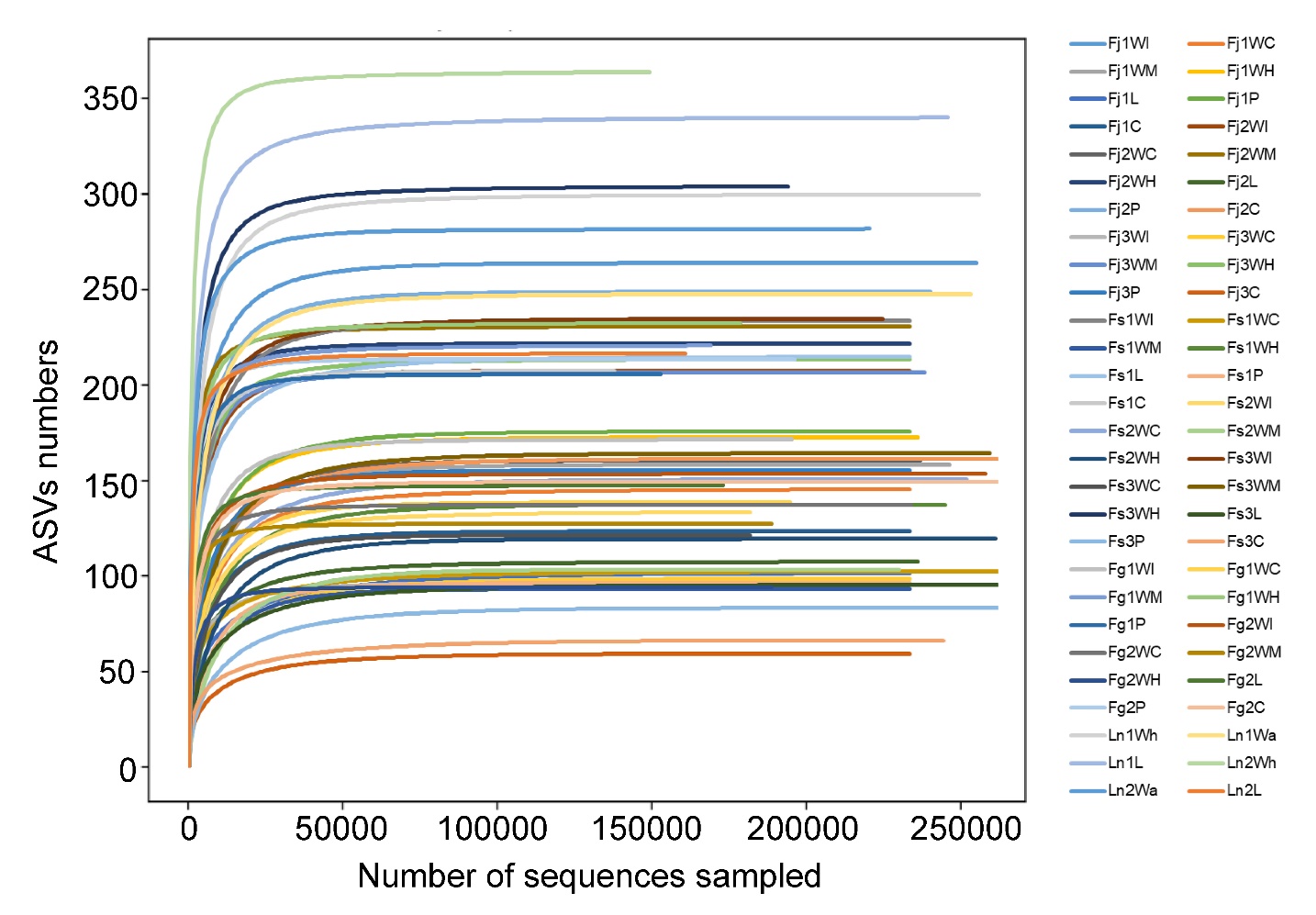


**Supplementary Figure 1.** Rarefaction curves of each sample of the four ant species.

**
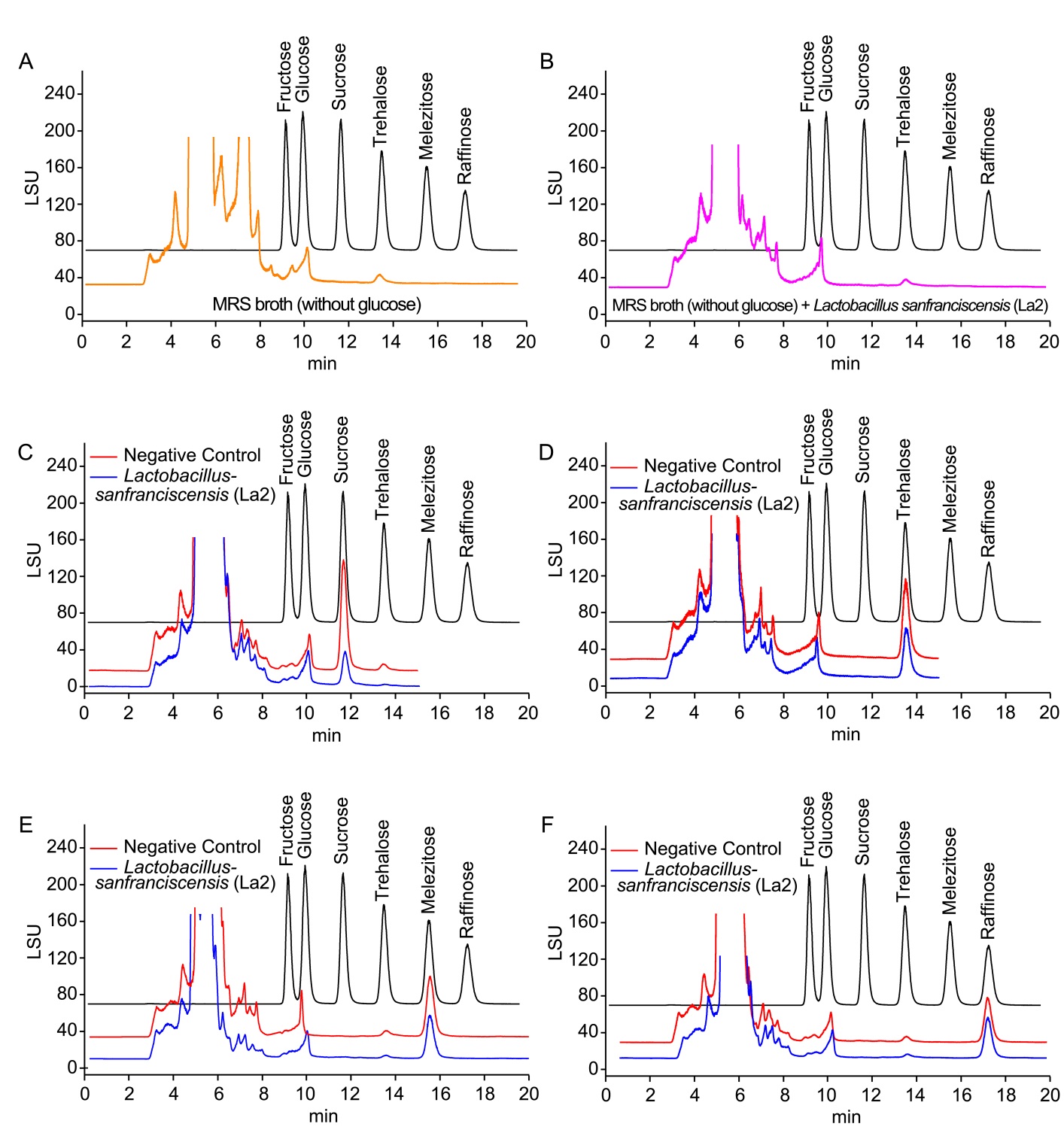
**

**Supplementary Figure 2.** High-Performance Liquid Chromatography–Evaporative Light-Scattering Detector (HPLC-ELSD) profile analysis of sugar catabolism ability of *Lactobacillus sanfranciscensis* (La2). Sucrose, trehalose, melezitose and raffinose are measured in light scattering units (LSU). **(A)** The sugar profile of MRS broth (without glucose). **(B)** The sugar profile of MRS broth (without glucose) supplemented with *L. sanfranciscensis* (La2). **(C-F)** The sugar profile of MRS broth (without glucose) supplemented with sucrose **(C)**, trehalose **(D)**, melezitose **(E)**, raffinose **(F)**. Using the boiled *L. sanfranciscensis* (La2) as negative control. The different sugar standard (20 μg of fructose, glucose, sucrose, trehalose, melezitose, and raffinose) was indicated in the graph.

**
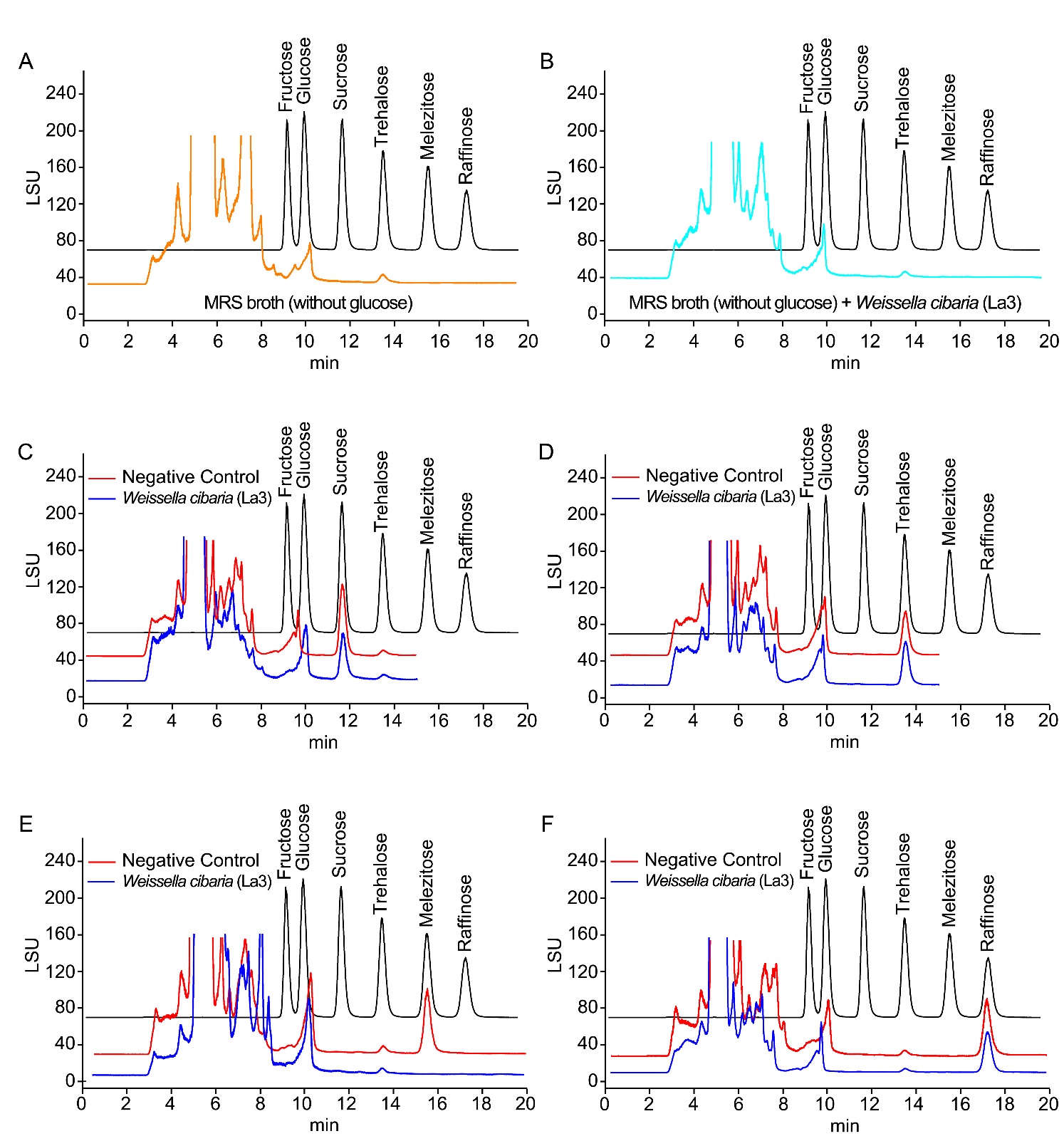
**

**Supplementary Figure 3.** High-Performance Liquid Chromatography–Evaporative Light-Scattering Detector (HPLC-ELSD) profile analysis of sugar catabolism ability of *Weissella cibaria* (La3). Sucrose, trehalose, melezitose and raffinose are measured in light scattering units (LSU). **(A)** The sugar profile of MRS broth (without glucose). **(B)** The sugar profile of MRS broth (without glucose) supplemented with *W. cibaria* (La3). **(C-F)** The sugar profile of MRS broth (without glucose) supplemented with sucrose **(C)**, trehalose **(D)**, melezitose **(E)**, raffinose **(F)**. Using the boiled *W. cibaria* (La3) as negative control. The different sugar standard (20 μg of fructose, glucose, sucrose, trehalose, melezitose, and raffinose) was indicated in the graph.

**Supplementary Table 1** Detailed information of *Lactobacillus* infection rates in different honeydew-feeding ants

| **Subfamily** | **Ant species** | **Number of workers** | **Number of infected individuals** | **Tree species** | **Sampling site** | **Longitude** | **Latitude** | **Altitude(m)** |
| --- | --- | --- | --- | --- | --- | --- | --- | --- |
| Formicinae | *Formica japonica* | 10 | 7 | *Ligustrum lucidum* | Xi'an City | 34.42 | 109.00 | 374 |
|  |  | 10 | 3 | *Populus davidiana* | Xi'an City | 34.42 | 109.00 | 374 |
|  |  | 10 | 8 | *Salix babylonica* | Xi'an City | 34.42 | 109.00 | 374 |
|  |  | 10 | 9 | *Paulownia fortunei* | Qian County | 34.47 | 108.21 | 560 |
|  |  | 10 | 9 | *Salix babylonica* | Taibai County | 34.21 | 107.14 | 1480 |
|  |  | 10 | 10 | *Toona sinensis* | Taibai County | 34.16 | 107.31 | 1836 |
|  |  | 10 | 10 | *Castanea mollissima* | Ningshan County | 33.21 | 108.26 | 1260 |
|  |  | 10 | 10 | *Toona sinensis* | Ningshan County | 33.47 | 108.50 | 2094 |
|  |  | 10 | 10 | *Aesculus chinensis* | Ningshan County | 33.33 | 108.31 | 1508 |
|  | *Formica glauca* | 10 | 10 | *Robinia pseudoacacia* | Xi'an City | 34.42 | 109.00 | 374 |
|  |  | 10 | 8 | *Chaenomeles sinensis* | Xi'an City | 34.15 | 108.58 | 410 |
|  |  | 10 | 1 | *Prunus persica* | Xi'an City | 34.18 | 108.93 | 430 |
|  |  | 10 | 0 | *Acer mono* | Xi'an City | 34.18 | 108.93 | 430 |
|  |  | 10 | 5 | *Robinia pseudoacacia* | Qian County | 34.47 | 108.21 | 560 |
|  |  | 10 | 8 | *Cedrus deodara* | Qian County | 34.47 | 108.21 | 560 |
|  |  | 10 | 9 | *Populus tomentosa* | Qian County | 34.57 | 108.23 | 763 |
|  |  | 10 | 9 | *Malus pumila* | Qian County | 34.30 | 108.10 | 620 |
|  |  | 10 | 6 | *Populus tomentosa* | Qian County | 34.30 | 108.10 | 620 |
|  | *Formica cunicularia* | 10 | 9 | *Salix babylonica* | Xi'an City | 34.42 | 109.00 | 374 |
|  |  | 10 | 6 | *Pyrus pyrifolia* | Xi'an City | 34.15 | 108.58 | 410 |
|  |  | 10 | 8 | *Punica granatum* | Xi'an City | 34.15 | 108.58 | 410 |
|  |  | 10 | 10 | *Acer mono* | Xi'an City | 34.18 | 108.93 | 430 |
|  |  | 10 | 7 | *Cedrus deodara* | Qian County | 34.47 | 108.21 | 560 |
|  |  | 10 | 10 | *Paulownia fortunei* | Qian County | 34.47 | 108.21 | 560 |
|  |  | 10 | 10 | *Populus tomentosa* | Qian County | 34.57 | 108.23 | 763 |
|  |  | 10 | 3 | *Ligustrum lucidum* | Qian County | 34.57 | 108.23 | 763 |
|  |  | 10 | 0 | *Prunus cerasifera* | Qian County | 34.57 | 108.23 | 763 |
|  |  | 10 | 9 | *Malus pumila* | Qian County | 34.30 | 108.10 | 620 |
|  |  | 10 | 8 | *Populus tomentosa* | Qian County | 34.30 | 108.10 | 620 |
|  | *Formica gagatoides* | 10 | 8 | *Picea asperata* | Ningshan County | 33.47 | 108.50 | 2094 |
|  | *Formica sanguinea* | 10 | 9 | *Salix babylonica* | Taibai County | 34.33 | 107.18 | 1839 |
|  | *Formica polyctena* | 10 | 10 | *Cinnamomum camphora* | Taibai County | 34.35 | 107.18 | 1839 |
|  | *Lasius niger* | 10 | 3 | *Ligustrum lucidum* | Xi'an City | 34.42 | 109.00 | 374 |
|  |  | 10 | 2 | *Populus tomentosa* | Xi'an City | 34.42 | 109.00 | 374 |
|  |  | 10 | 4 | *Salix babylonica* | Xi'an City | 34.42 | 109.00 | 374 |
|  |  | 10 | 6 | *Chaenomeles sinensis* | Xi'an City | 34.15 | 108.58 | 410 |
|  |  | 10 | 0 | *Pyrus pyrifolia* | Xi'an City | 34.15 | 108.58 | 410 |
|  |  | 10 | 0 | *Punica granatum* | Xi'an City | 34.15 | 108.58 | 410 |
|  |  | 10 | 0 | *Acer mono* | Xi'an City | 34.18 | 108.93 | 430 |
|  |  | 10 | 0 | *Salix babylonica* | Taibai County | 34.21 | 107.14 | 1480 |
|  |  | 10 | 4 | *Larix gmelinii* | Taibai County | 34.16 | 107.31 | 1836 |
|  |  | 10 | 0 | *Populus tomentosa* | Taibai County | 34.16 | 107.31 | 1836 |
|  |  | 10 | 8 | *Prunus cerasifera* | Taibai County | 34.16 | 107.31 | 1827 |
|  |  | 10 | 3 | *Castanea mollissima* | Ningshan County | 33.21 | 108.26 | 1260 |
|  |  | 10 | 7 | *Castanea mollissima* | Ningshan County | 33.21 | 108.26 | 1260 |
|  |  | 10 | 9 | *Aesculus chinensis* | Ningshan County | 33.33 | 108.31 | 1260 |
|  | *Lasius fuliginosus* | 10 | 10 | *Juglans regia* | Ningshan County | 33.40 | 108.37 | 1074 |
|  | *Camponotus japonicus* | 10 | 5 | *Robinia pseudoacacia* | Qian County | 34.47 | 108.21 | 560 |
|  |  | 10 | 4 | *Cedrus deodara* | Qian County | 34.47 | 108.21 | 560 |
|  |  | 10 | 2 | *Populus tomentosa* | Qian County | 34.57 | 108.23 | 763 |
|  |  | 10 | 3 | *Malus pumila* | Qian County | 34.30 | 108.10 | 620 |
|  |  | 10 | 6 | *Malus pumila* | Qian County | 34.30 | 108.10 | 620 |
|  |  | 10 | 8 | *Populus tomentosa* | Qian County | 34.30 | 108.10 | 620 |
|  |  | 10 | 2 | *Salix babylonica* | Taibai County | 34.21 | 107.14 | 1480 |
|  | *Camponotus obscuripes* | 10 | 6 | *Larix gmelini* | Taibai County | 34.15845 | 107.3105 | 1836 |
| Myrmicinae | *Pristomyrmex pungens* | 10 | 10 | *Paulownia fortunei* | Ningshan County | 33.34115 | 108.3113 | 772 |
|  | *Crematogaster vagula* | 10 | 4 | *Ligustrum lucidum* | Xi'an City | 34.41843 | 108.9953 | 374 |
|  |  | 10 | 3 | *Prunus cerasifera* | Xi'an City | 34.17861 | 108.9253 | 430 |
|  |  | 10 | 0 | *Acer mono* | Xi'an City | 34.17861 | 108.9253 | 430 |
|  |  | 10 | 8 | *Paulownia fortunei* | Qian County | 34.46737 | 108.215 | 560 |
|  |  | 10 | 1 | *Populus tomentosa* | Qian County | 34.56654 | 108.2251 | 763 |
|  |  | 10 | 0 | *Ligustrum lucidum* | Qian County | 34.56654 | 108.2251 | 763 |
|  | *Crematogaster biroi* | 10 | 0 | *Malus pumila* | Qian County | 34.3019 | 108.1041 | 620 |
|  | *Crematogaster artifex* | 10 | 0 | *Phyllostachys edulis* | Ningshan County | 33.34115 | 108.3113 | 772 |
|  | *Dolichoderus sibiricus* | 10 | 0 | *Paulownia fortunei* | Qian County | 34.46737 | 108.215 | 560 |

**Supplementary Table 2** List of lactic acid bacteria strains isolated from honeydew-feeding ant workers, distributed by ant species

| Ant species | Colony | Tree species | Replicate number | Isolated sources and numbers | | | | | | Strains of lactic acid bacteria |
| --- | --- | --- | --- | --- | --- | --- | --- | --- | --- | --- |
|  |  |  |  | IBPs | Crops | Midguts | Hindguts | Heads | Gasters |  |
| *Formica cunicularia* | J1 | *Malus halliana* | Replicate 1 | 1 | 508 | 0 | 0 | / | / | La1, La2, La3, La4, La5, La6, La7, La8, La10, La11, La12, La13 |
|  |  |  | Replicate 2 | 896 | 2600 | 0 | 0 | / | / |  |
|  |  |  | Replicate 3 | 0 | 720 | 0 | 0 | / | / |  |
|  | J2 | *Cedrus deodara* | Replicate 1 | 1680 | 1220 | 0 | 0 | / | / | the same as above |
|  |  |  | Replicate 2 | 4 | 12 | 0 | 0 | / | / |  |
|  |  |  | Replicate 3 | 2040 | 12 | 0 | 0 | / | / |  |
|  | LA | *Koelreuteria paniculata* | Replicate 1 | 1 | 60 | 0 | 0 | / | / | the same as above |
|  |  |  | Replicate 2 | 28 | 17 | 0 | 0 | / | / |  |
|  |  |  | Replicate 3 | 1 | 230 | 0 | 0 | / | / |  |
|  | QA | *Aesculus chinensis* | Replicate 1 | 0 | 13 | 0 | 0 | / | / | La1, La2, La3, La4, La5, La6, La7, La8, La9, La10, La11, La12, La13 |
|  |  |  | Replicate 2 | 5 | 13 | 0 | 0 | / | / |  |
|  |  |  | Replicate 3 | 0 | 960 | 0 | 0 | / | / |  |
| *Formica japonica* | XC | *Cedrus deodara* | Replicate 1 | 1 | 1800 | 0 | 0 | / | / | La1, La2, La3, La4, La5, La6, La7, La8, La10, La11, La12, La13 |
|  |  |  | Replicate 2 | 0 | 0 | 0 | 0 | / | / |  |
|  |  |  | Replicate 3 | 0 | 0 | 0 | 0 | / | / |  |
|  | CC | *Magnolia denudata* | Replicate 1 | 2 | 87 | 0 | 0 | / | / | the same as above |
|  |  |  | Replicate 2 | 12 | 0 | 0 | 0 | / | / |  |
|  |  |  | Replicate 3 | 5 | 0 | 0 | 0 | / | / |  |
|  | YC | *Malus halliana* | Replicate 1 | 6 | 2 | 0 | 0 | / | / | the same as above |
|  |  |  | Replicate 2 | 1 | 530 | 0 | 0 | / | / |  |
|  |  |  | Replicate 3 | 4 | 0 | 0 | 0 | / | / |  |
| *Formica fusca* | L1 | *Sophora japonica* | Replicate 1 | 95 | 0 | 0 | 0 | / | / | the same as above |
|  |  |  | Replicate 2 | 0 | 1 | 0 | 0 | / | / |  |
|  |  |  | Replicate 3 | 2760 | 105 | 0 | 0 | / | / |  |
| *Lasius niger* | XB | *Cedrus deodara* | Replicate 1 | / | / | / | / | 17 | 9 | La1, La11, La14 |
|  |  |  | Replicate 2 | / | / | / | / | 0 | 0 |  |
|  |  |  | Replicate 3 | / | / | / | / | 0 | 0 |  |
|  | TB | *Amygdalus persica* | Replicate 1 | / | / | / | / | 1 | 0 | / |
|  |  |  | Replicate 2 | / | / | / | / | 0 | 0 |  |
|  |  |  | Replicate 3 | / | / | / | / | 0 | 0 |  |
|  | MB | *Hibiscus syriacus* | Replicate 1 | / | / | / | / | 0 | 0 | / |
|  |  |  | Replicate 2 | / | / | / | / | 0 | 0 |  |
|  |  |  | Replicate 3 | / | / | / | / | 0 | 0 |  |
|  | HB | *Sophora japonica* | Replicate 1 | / | / | / | / | 0 | 0 | / |
|  |  |  | Replicate 2 | / | / | / | / | 0 | 0 |  |
|  |  |  | Replicate 3 | / | / | / | / | 0 | 0 |  |
|  | LB | *Koelreuteria paniculata* | Replicate 1 | / | / | / | / | 0 | 0 | / |
|  |  |  | Replicate 2 | / | / | / | / | 0 | 0 |  |
|  |  |  | Replicate 3 | / | / | / | / | 0 | 0 |  |
|  | ZB | *Koelreuteria paniculata* | Replicate 1 | / | / | / | / | 2 | 4 | La1, La7 |
|  |  |  | Replicate 2 | / | / | / | / | 1 | 3 |  |
|  |  |  | Replicate 3 | / | / | / | / | 1 | 3 |  |
|  | YSE | *Malus pumila* | Replicate 1 | / | / | / | / | 0 | 0 | / |
|  |  |  | Replicate 2 | / | / | / | / | 0 | 0 |  |
|  |  |  | Replicate 3 | / | / | / | / | 0 | 0 |  |
|  | YK | *Ulmus pumila* | Replicate 1 | / | / | / | / | 0 | 0 | / |
|  |  |  | Replicate 2 | / | / | / | / | 1 | 0 |  |
|  |  |  | Replicate 3 | / | / | / | / | 0 | 0 |  |
| *Pristomyrmex pungens* | PB | *Eriobotrya japonica* | Replicate 1 | / | / | / | / | 0 | 0 | / |
|  |  |  | Replicate 2 | / | / | / | / | 0 | 0 |  |
|  |  |  | Replicate 3 | / | / | / | / | 1 | 0 |  |
|  | H1 | *Malus pumila* | Replicate 1 | / | / | / | / | 0 | 0 | / |
|  |  |  | Replicate 2 | / | / | / | / | 0 | 0 |  |
|  |  |  | Replicate 3 | / | / | / | / | 0 | 0 |  |
| *Crematogaster vagula* | HH | *Albizzia julibrissin* | Replicate 1 | / | / | / | / | 0 | 0 | La1, La2, La3, La15 |
|  |  |  | Replicate 2 | / | / | / | / | 1440 | 0 |  |
|  |  |  | Replicate 3 | / | / | / | / | 0 | 0 |  |
| *Dolichoderus sibiricus* | TD | *Amygdalus persica* | Replicate 1 | / | / | / | / | 0 | 0 | La1, La2 |
|  |  |  | Replicate 2 | / | / | / | / | 160 | 0 |  |
|  |  |  | Replicate 3 | / | / | / | / | 0 | 0 |  |
